# Competing for Iron: Duplication and Amplification of the *isd* Locus in *Staphylococcus lugdunensis* HKU09-01 Provides a Competitive Advantage to Overcome Nutritional Limitation

**DOI:** 10.1371/journal.pgen.1006246

**Published:** 2016-08-30

**Authors:** Simon Heilbronner, Ian R. Monk, Jeremy R. Brozyna, David E. Heinrichs, Eric P. Skaar, Andreas Peschel, Timothy J. Foster

**Affiliations:** 1 Microbiology Department, Trinity College, Dublin, Ireland; 2 Infection Biology, Interfaculty Institute of Microbiology and Infection Medicine, University of Tübingen, Tübingen, Germany; 3 Department of Microbiology and Immunology, University of Melbourne, Doherty Institute for Infection and Immunity, Parkville, Victoria, Australia; 4 Department of Microbiology and Immunology, University of Western Ontario, London, Ontario, Canada; 5 Department of Pathology, Microbiology, and Immunology, Vanderbilt University School of Medicine and Tennessee Valley Healthcare Systems, US Department of Veterans Affairs, Nashville, Tennessee, United States of America; The University of Texas Health Science Center at Houston, UNITED STATES

## Abstract

*Staphylococcus lugdunensis* is a coagulase negative bacterial pathogen that is particularly associated with severe cases of infectious endocarditis. Unique amongst the coagulase-negative staphylococci, *S*. *lugdunensis* harbors an iron regulated surface determinant locus (*isd*). This locus facilitates the acquisition of heme as a source of nutrient iron during infection and allows iron limitation caused by “nutritional immunity” to be overcome. The *isd* locus is duplicated in *S*. *lugdunensis* HKU09-01 and we show here that the duplication is intrinsically unstable and undergoes accordion-like amplification and segregation leading to extensive *isd* copy number variation. Amplification of the locus increased the level of expression of Isd proteins and improved binding of hemoglobin to the cell surface of *S*. *lugdunensis*. Furthermore, Isd overexpression provided an advantage when strains were competing for a limited amount of hemoglobin as the sole source of iron. Gene duplications and amplifications (GDA) are events of fundamental importance for bacterial evolution and are frequently associated with antibiotic resistance in many species. As such, GDAs are regarded as evolutionary adaptions to novel selective pressures in hostile environments pointing towards a special importance of *isd* for *S*. *lugdunensis*. For the first time we show an example of a GDA that involves a virulence factor of a Gram-positive pathogen and link the GDA directly to a competitive advantage when the bacteria were struggling with selective pressures mimicking “nutritional immunity”.

## Author Summary

Sometimes changing environmental conditions force bacteria to boost protein expression above the level that can be achieved by transcriptional or translational control. Gene duplication and amplification (GDA) represents a simple and effective means to augment protein expression and is frequently associated with the development of resistance against antibacterial agents. As such GDAs can be seen as recent evolutionary adaptions towards novel selective pressures within the environment. We describe herein the GDA of the *isd* locus in a clinical isolate of *Staphylococcus lugdunensis* a bacterial species frequently associated with infectious endocarditis. This amplification made individual lineages significantly more successful when competing for the trace element iron supplied in the form of hemoglobin (hb). Human body fluids are actively depleted of iron to prevent bacterial proliferation and hb represents the most abundant iron source during infection. This deepens our understanding about adaption processes of pathogens and demonstrates how selective pressures drive the evolution of pathogens to become more successful. This study suggests that investigating GDAs in clinical isolates can help to identify chromosomal regions that are of special importance for bacterial fitness under changing environmental conditions. The gene products can therefore represent attractive targets for novel antibiotics supporting the natural immune defenses by targeting the vulnerable spots of the bacterial anti-immunity strategies.

## Introduction

Bacterial pathogens possess a tremendous ability to adapt to changing environmental conditions. This is especially noticeable within the hospital setting where strong artificial selective pressures such as antibiotics and disinfectants drive the evolution of pathogens to develop resistance to these agents. In contrast, during invasive disease a plethora of immune strategies ranging from cellular defences and antimicrobial molecules to nutrient limitation apply natural selective pressures that might drive the evolution of organisms to become more pathogenic.

Commonly encountered mechanisms of acquired resistance to antibacterial drugs or increased virulence involve horizontal gene transfer or spontaneous mutations. Another mechanism is to acquire gene duplications and amplifications (GDA), a mechanism based on the RecA-dependent accordion-like expansion and contraction of repetitive DNA sequences leading to gene dosage effects. GDAs are most commonly associated with drug resistance, often by increasing the expression of minor resistance determinants [1]. However, GDAs should also hold the potential to enhance bacterial virulence or survival within the host, for example by increasing virulence gene expression, a possibility that has been neglected in the research community. This disregard might be due to the fact that GDAs are in general difficult to detect using next generation sequencing because of the sequence identity of the individual repeats (up to 100% on DNA level). Furthermore, gene dosage effects are difficult to study and GDAs are frequently transient and lost quickly when the selective pressure is relieved making experimental investigation challenging. Accordingly, very little is known about the natural occurrence of GDAs in the chromosomes of pathogens and about how they can promote virulence, immune evasion or growth under *in vivo*-like conditions.

Although *S*. *lugdunensis* belongs to the coagulase negative staphylococci (CoNS), in many ways it behaves more like the coagulase positive *S*. *aureus* than other CoNS, including having an apparently elevated degree of virulence. As a result, *S*. *lugdunensis* has been referred to as a wolf in sheep’s clothing [2]. Apart from skin and soft tissue infections, *S*. *lugdunensis* is particularly associated with an aggressive form of infective endocarditis (IE) with 70% of the patients requiring surgery compared to 37% of patients suffering from *S*. *aureus* IE [3].

Iron (Fe3+/Fe2+) is a scarce nutrient during invasive disease since body fluids are actively depleted of free iron and other divalent metals by the host, in order to prevent bacterial growth. This process has been referred to as “nutritional immunity” [4]. A noteworthy characteristic of the genome sequence of *S*. *lugdunensis* [5] is an iron-dependent surface determinant (*isd*) locus. *S*. *aureus* is the only other staphylococcal species known to harbor an *isd* locus where it facilitates the acquisition of heme from hemoglobin as a source of nutrient iron. This allows the bacteria to overcome iron restriction during host colonization and invasive disease [6]. Interestingly, the *isd* operon was found to be tandemly duplicated in the *S*. *lugdunensis* clinical isolate HKU09-01 [5, 7].

Here we demonstrated that this duplication undergoes constant RecA-dependent amplification and segregation, leading to drastic changes of the *isd* expression levels. Duplication and amplification of *isd* improved the hemoglobin binding capacity and provided a significant advantage when strains were competing for hemoglobin as a source for nutrient iron. For the first time we demonstrate the mechanism of GDA acting on a virulence factor of a Gram-positive pathogen serving as a means to improve the competitive fitness of the bacteria in order to overcome nutrient limitation mimicking “nutritional immunity”.

## Results

### The *isd* locus is tandemly duplicated in *S*. *lugdunensis* HKU09-01

The comparative analysis of the *S*. *lugdunensis* N920143 and HKU09-01 genome sequences revealed a 32-kb duplication (S1 Fig) in the latter strain. In N920143 (single copy of the region) the orfs SLUG_00860 and SLUG_01140 possess a short stretch of identity (19 nucleotides in length with only one mismatch). Unequal recombination must have occurred at this site in HKU09-01 (SLGD_00056 and SLGD_00116), thereby duplicating the ~16kb *isd* operon as well as the genes encoding the SirABC transporter, a MarR family transcriptional regulator, and ten genes encoding proteins that are putatively involved in general metabolism and substrate import/export. Furthermore, the recombination event created a unique hybrid gene in HKU09-01 (SLGD_00087) comprising the 5’ end of SLGD_00058 and the 3’ end of SLGD_00116.

**Fig 1:**
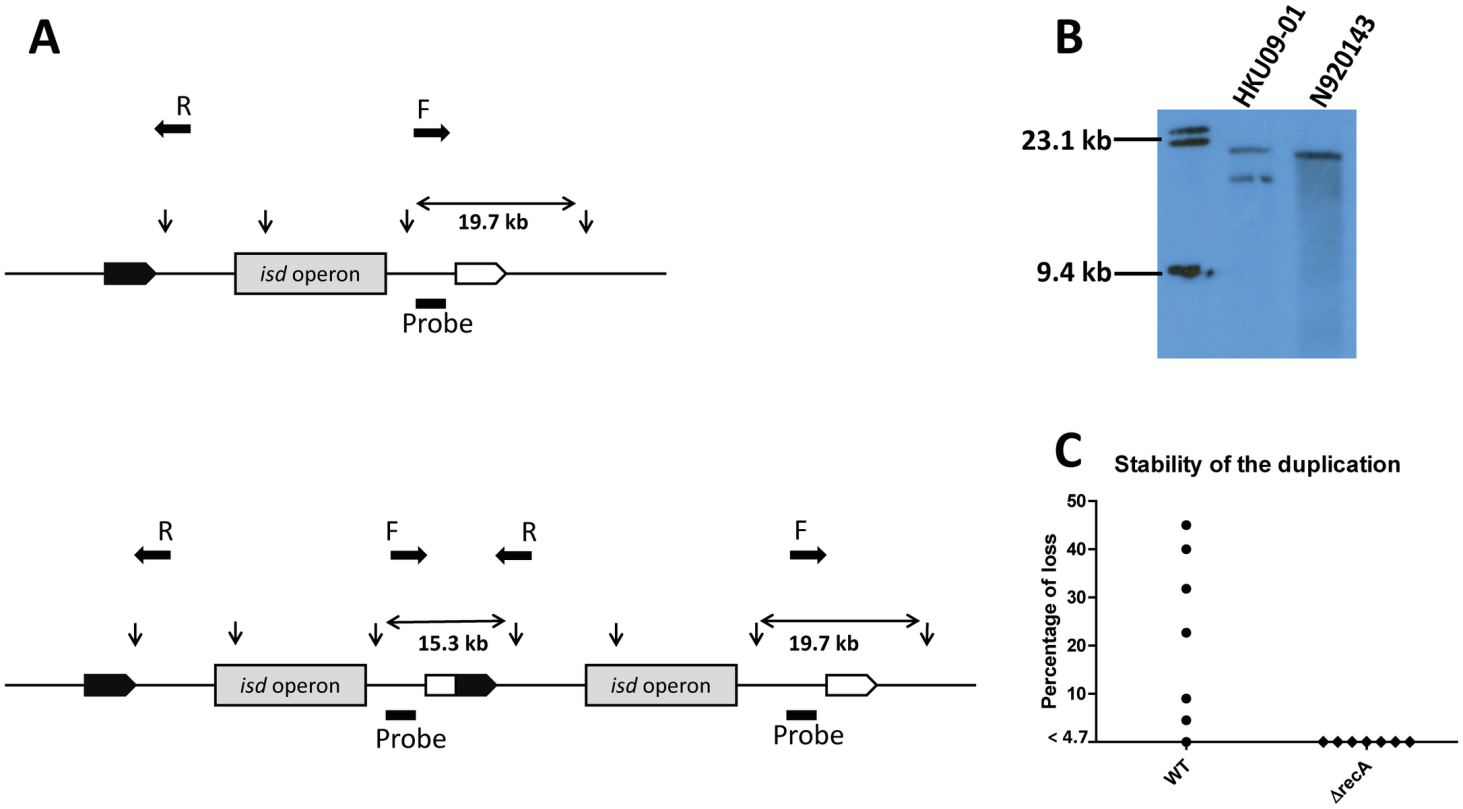
Duplication of *isd* in HKU09-01. (A) Schematic diagram of the ApaLI restriction sites in the single region of N920143 and the duplication in HKU09-01. Restriction sites are indicated by vertical arrows. The binding site of the DIG-labelled probe is indicated by the black dash. Predicted sizes of the fragments recognized by the probe are indicated. Primer binding sites and amplification direction (F and R) for the rapid screening for the duplication are indicated by horizontal filled arrows. (B) Results of the Southern blot. Chromosomal DNA of *S*. *lugdunensis* strains N920143 and HKU09-01 were digested with ApaLI and separated by electrophoresis. The DNA fragments were subsequently denatured, blotted onto a nylon membrane and hybridized with the DIG-labelled probe. Hybridization was detected using anti-DIG-Fab fragments conjugated to alkaline phosphatase. (C) HKU09-01 (wild-type and Δ*recA* mutant) cultures were plated out and 22 colonies were screened for the presence of the duplication using primer F/R indicated in A. The frequency of loss of the duplication of seven independent cultures is shown.

To validate the sequence data, we performed a Southern blot experiment using the restriction enzyme ApaLI to cleave genomic DNA and a digoxigenin-labelled probe. The presence of the duplication created a distinct restriction pattern and an additional binding site for the probe (Fig 1A and 1B). The detection of a ~15.3 kb and a ~19.7 kb fragment was consistent with the presence of the duplication in HKU09-01, while a single ~19.7 kb fragment confirmed a single copy in N920143.

### Stability of the duplication is RecA dependent

It is recognized that duplications give rise to a high frequency of RecA-mediated recombination events due to the long stretches of homology. This leads either to amplification of the locus, or to loss of the duplication (segregation). In order to investigate whether these processes happen in HKU09-01, a *recA* deletion mutation was isolated in HKU09-01 by allelic replacement. RecA-deficient mutants of *S*. *lugdunensis* strains have a reduced growth rate and are hypersensitive to DNA damage induced by UV light [8]. These phenotypes were confirmed for HKU09-01 Δ*recA*.

A PCR colony screening method was developed for the rapid discrimination of HKU09-01 colonies harbouring or lacking the duplication. To this end, primers were used facing the boundaries of the duplication which amplified a product only if the duplication was retained (see Fig 1A). Individual colonies arising from independent overnight cultures were screened for the presence of the duplication (Fig 1C). A variable percentage (up to 45%) of colonies in the RecA^+^ background showed loss of the duplication. In contrast, RecA deficiency resulted in stabilisation of the duplication, confirming RecA as the major driver of segregation.

### Amplification of *isd*

**Fig 2:**
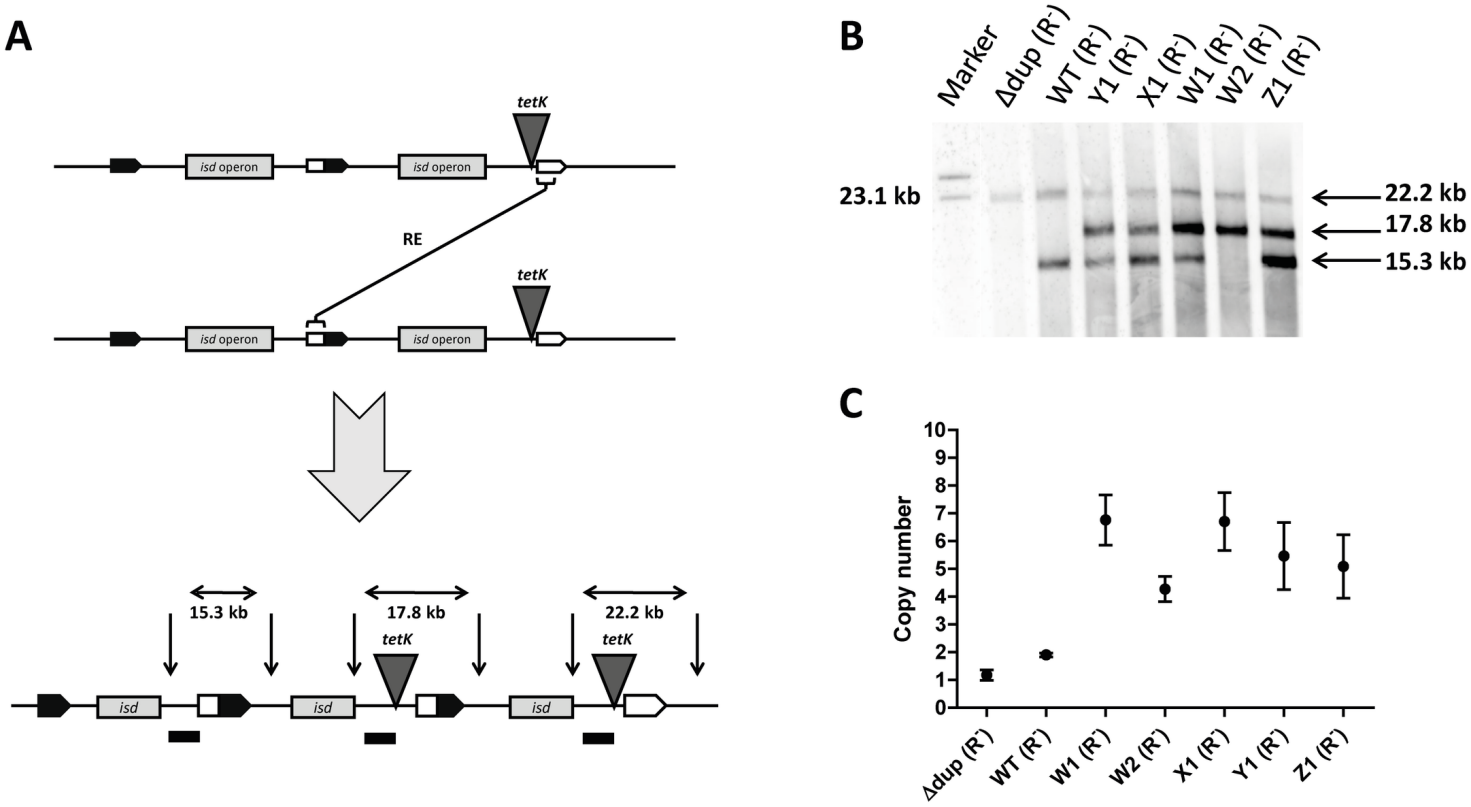
Amplification of *isd*. (A) Schematic diagram of the recombination event (RE) leading to the different numbers of fragments labelled in the Southern blot experiment. ApaLI restriction sites are indicated by vertical arrows. The binding site of the DIG-labelled probe is indicated by the black dash. Predicted sizes of the fragments recognized by the probe are indicated. (B) Results of the Southern blot. Chromosomal DNA of HKU::*tetK* strains (*ΔrecA*) with different tetracycline resistance levels were digested with ApaLI and separated by electrophoresis. The DNA fragments were subsequently denatured, blotted onto a nylon membrane and hybridized with the DIG-labelled probe. Hybridization was detected using anti-DIG-Fab fragments conjugated to alkaline phosphatase. Y1, X1, W1, W2 and Z1 designate strains with different colony sizes on 8 μg/ml Tc. (C) qPCR experiment to determine the *isd* copy number. Known concentrations of N920143 DNA (one copy of *isd*) were used to create the standard curves for *isdJ* and *ori*. Relative amounts of template *ori* and *isdJ* for each strain (all *ΔrecA*) were measured. The value for *ori* was set to 1 and the template amount of *isdJ* was expressed in relation to this value, thereby giving the copy number of *isdJ* in the chromosome of each strain. The mean and SD of three experiments is shown.

The model for GDA indicates that as soon as a duplication is established segregation and amplification occur hand in hand. A well studied effect of GDAs is the increase in the level of antibiotic resistance when the gene dosage of the resistance determinant is increased. Therefore we integrated a tetracycline (Tc) resistance determinant (*tetK*) at the 3’ end of the duplication using allelic exchange (S2 Fig) so that amplification of the duplicated region included *tetK*. The *tetk* gene confers low level Tc resistance (Tc^R^) when present in a single copy but high level resistance when present in multiple copies [9]. This enabled us to trace any subsequent amplification by measuring the Tc resistance level and by Southern blotting. The *tetK* insertion site was located on one of the fragments detected by the probe which therefore increased in size (19.7 kb fragment to 22.2 kb) (S2 Fig). Due to this location of *tetK*, amplification created a third fragment of 17.8 kb that could be picked up by the probe (Fig 2A). The strain HKU::*tetK* was resistant to 4 μg/ml Tc but was unable to grow on 8 μg/ml Tc. However, after growth in liquid culture (without Tc) many colonies arose on 8 μg/ml Tc with colony sizes ranging from ~0.1 mm to ~1.5 mm suggesting that they harboured different copy numbers of *tetK*. Several colonies were picked and a *recA* mutation was introduced by allelic exchange to stabilize the copy number. Southern blotting (Fig 2B) showed that the strain HKU::*tetK* Δdup (*tetK* integrated into a strain isolated by the PCR screen above) had lost the duplication, indicated by a single 22.2 kb fragment. The HKU::*tetK* derivative had maintained 2 copies (~15,3 kb and a ~22,2 kb fragments) while all strains with increased Tc resistance showed an additional band of ~17,8 kb confirming the amplification of *isd* and *tetK*. Interestingly the strain W2 had lost the ~15,3 kb fragment suggesting that amplification occurred prior to a segregation event that deleted the 5’ end of the gene array. However, both methods are not suited to directly determine the *isd* copy number. Besides amplification of *tetK*, Tc resistance levels could also be influenced by spontaneous mutations within *tetK* or elsewhere in the chromosome and measurement of the intensity of bands in Southern Blot experiments is inaccurate due to long incubation times and the enzymatic detection method. Furthermore, the method might be misleading since the probe does not label *isd* directly but a fragment upstream of the *isd* locus. Therefore, we decided to use quantitative PCR experiments for an accurate determination of the *isd* copy number. The *isd* copy number of the strains was determined comparing the relative template amount of the origin of replication (one copy per cell) to the relative template amount of *isdJ* within the *isd* operon. The results confirmed that strains HKU::*tetK* Δdup and HKU::*tetK* possessed one and two copies of *isd*, respectively. In contrast, the strains W1, W2, X1, Y1, Z1 with increased Tc resistance harboured 7, 4, 7, 5, 5 copies of *isd*, respectively (Fig 2C).

To construct a control for phenotypic characterisation we created the strain HKU::*tetK* Δ*isd* that had lost the duplication (due to segregation) and then the entire *isd* locus (*isdB* to *isdP*) was deleted by allelic exchange. For simplicity strains were designated according to their *isd* copy number: isd-0 (HKU::*tetK Δisd*), isd-1 (HKU::*tetK* Δdup), isd-2 (HKU::*tetK*) and isd-7 (X1) (S3 Fig). Importantly, all these strains harboured a Δ*recA* mutation to prevent further recombination.

### Isd protein expression correlates with *isd* copy number

**Fig 3:**
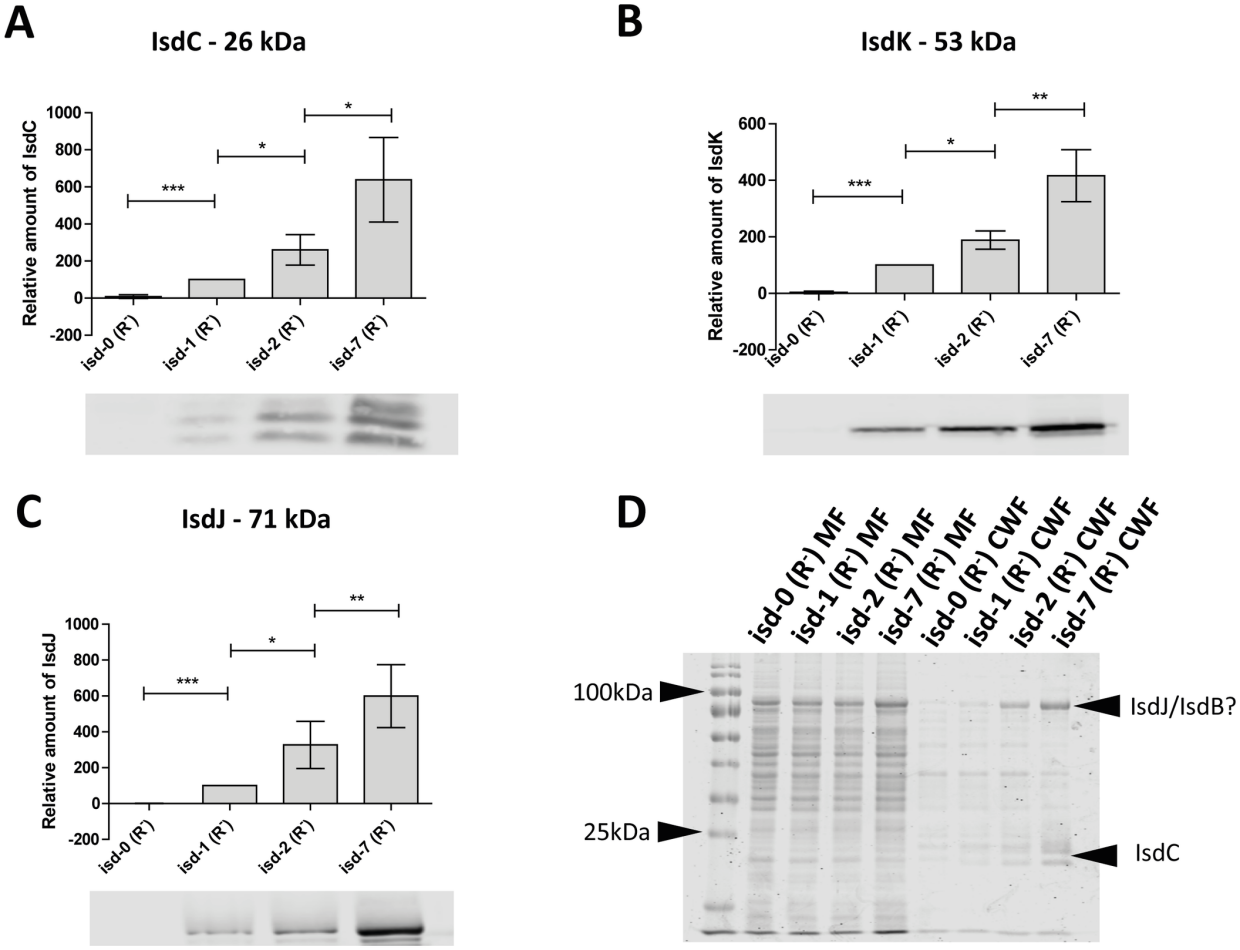
Isd protein expression. Isd copy number variants (*ΔrecA*) were grown in RPMI with 0,5 mM bipiridyl, adjusted to OD_578_ = 5 and the cell wall was digested with lysostaphin and mutanolysin in the presence of 500 mM sucrose to stabilize the protoplasts. (A,B,C)–Western Blot: Cell wall and membrane fractions were separated by SDS-PAGE and blotted onto a PVDF membrane. Isd proteins were detected using specific rabbit serum followed by goat anti-rabbit IgG DYLight 800. Fluorescent signals were quantified using Li-Core infrared technology. Absolute values measured for isd-1 were set to 100% and values obtained for the other strains were expressed in relation to this. The mean and SD of four independent experiments is shown. Statistical evaluation was performed using a paired two tailed t-test. P-values <0.05 were regarded as significant and are indicated by *. *** indicate P-values of <0.0001. IsdK was measured in the membrane fraction, IsdJ in the cell wall fraction and IsdC in the cell wall fraction. (D) Loading control: Each experiment was controlled by loading a part of the sample used for Western blotting on a second acrylamide gel. Cell wall and membrane fractions were separated by SDS-PAGE and stained with Coomassie blue. A representative gel is shown. Isd proteins predicted according to their size are indicated. MF- membrane fraction, CWF—cell wall fraction.

The variation of gene copy number suggested that the strains might express different amounts of Isd proteins. In order to quantify the expression of Isd, strains were grown in iron-restricted medium to induce expression of the locus. Cell fractionation was performed and Isd proteins within cell wall and membrane fractions were analysed by Western immunoblotting. The results showed that the increase in *isd* copy number correlated with an increase in the amount of Isd protein present (Fig 3A–3C). Loading was controlled using Coomassie staining (Fig 3D). Interestingly the increase in the level of IsdJ/B and IsdC (predicted by protein size) could also be observed in the Coomassie-stained cell wall fractions demonstrating the strong effect of the amplification. Overall protein levels confirmed equal loading.

**Fig 4:**
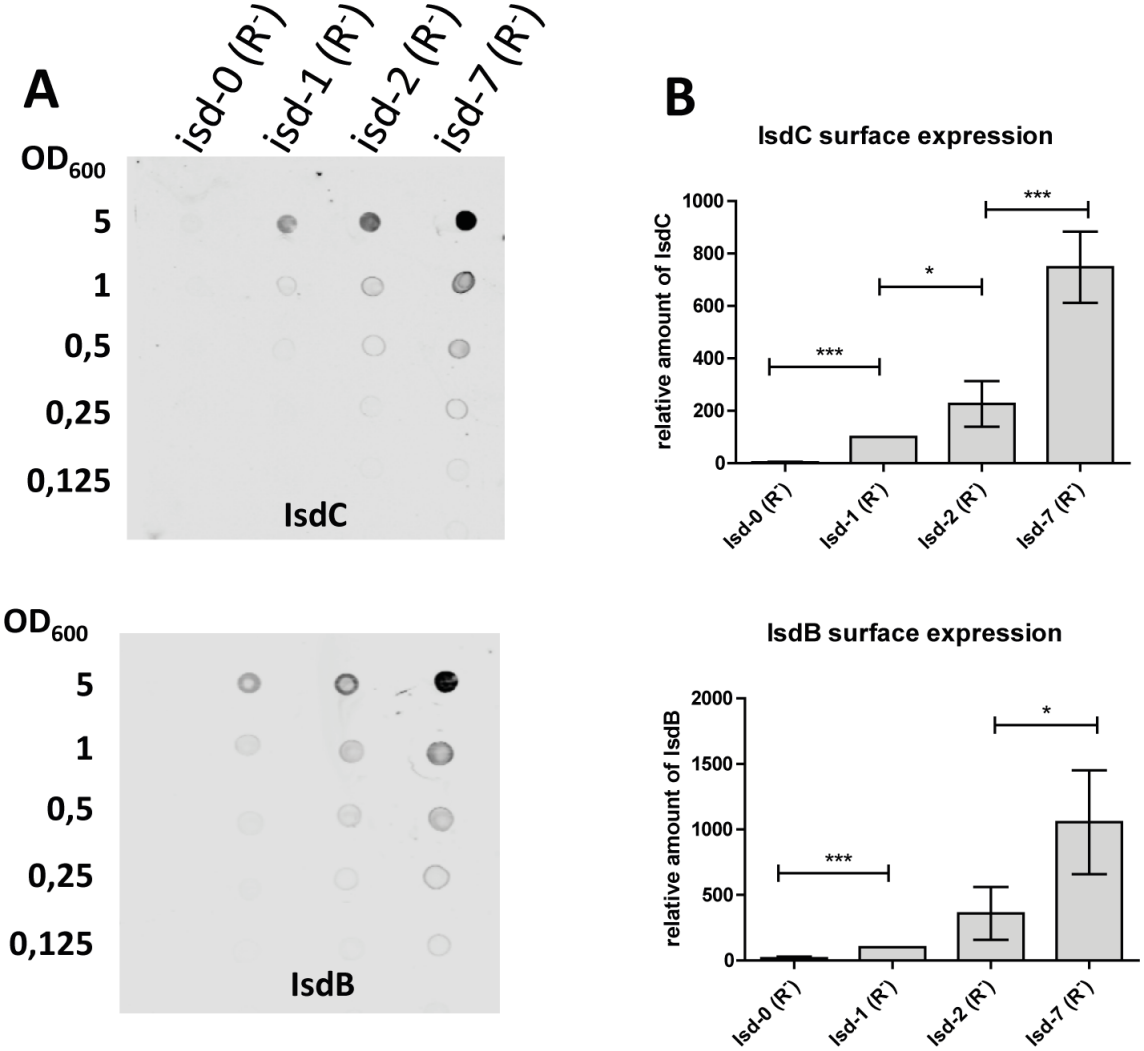
Isd surface expression. Whole cell immunoblot: Overnight cultures of *isd* copy number variants (*ΔrecA*) were grown in RPMI, adjusted to an OD_578_ = 5 and doubling dilutions were spotted on the membrane. Isd proteins were detected with specific rabbit serum followed by goat anti-rabbit IgG DYLight 800. Fluorescence intensity was measured quantitatively using Li-Core infrared detection. (A) Upper and lower panels show representative blots for IsdC and IsdB, respectively. (B) Statistical evaluation: Absolute values measured for isd-1 were set to 100% and values obtained for the other strains were expressed in relation to this. The mean and SD of five independent experiments is shown. Statistical evaluation was performed using a paired two tailed t-test. P-values of <0.05 were regarded as significant and are indicated by*. ** indicate P-values of <0.01 and *** indicate P-values of <0.0001.

Some Isd proteins are known to be directly exposed on the cell surface while others seem to be buried within the peptidoglycan [10]. We performed whole cell immunoblotting to determine whether the level of protein exposed on the surface of cells differed between the copy number variants. In fact, the level of IsdC and IsdB increased according to the *isd* copy number (Fig 4). Although IsdB was detected on the surface of cells, we failed to detect the protein in cell fractionations of the copy number variants using infra-red technology. Conversely, we were able to detect IsdB using conventional HRP detection and long exposure times (S4 Fig). However, due to the limited capacity of this detection method to achieve reliable quantifications, these data were only shown qualitatively.

### Amplification of *isd* improves the binding of hemoglobin to the cell surface

**Fig 5:**
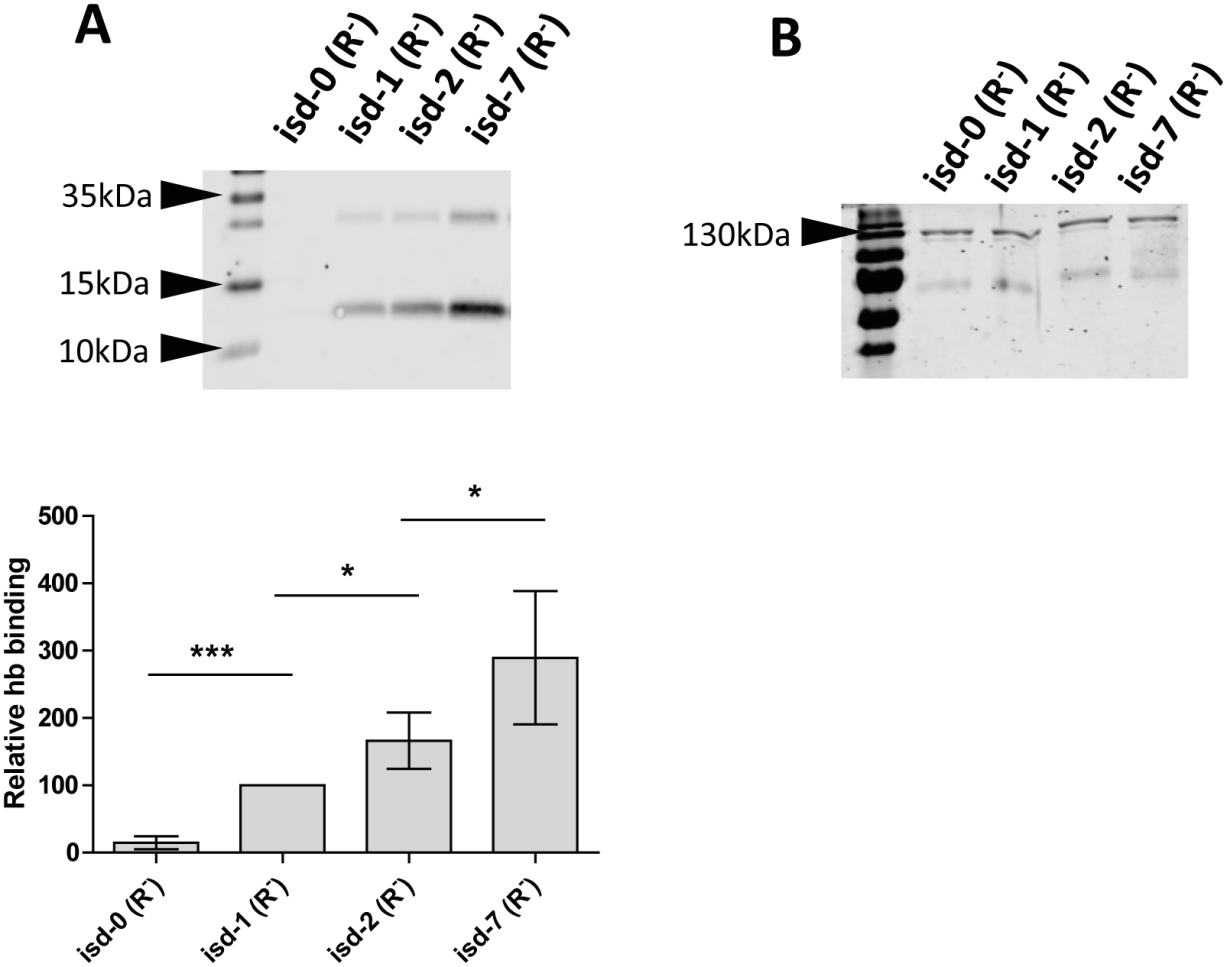
Binding of hemoglobin to the cell surface. Isd copy number variants (*ΔrecA*) were grown in RPMI with 0,5mM bipiridyl, adjusted to OD_578_ = 2 and incubated with 10 μg/ml human hemoglobin (hb). After washing cell-surface bound hb was released by boiling and supernatants were separated by SDS-PAGE. Proteins were blotted onto a PVDF membrane. (A) Human hb was detected using specific rabbit serum followed by goat anti-rabbit IgG DYLight 800. Fluorescence intensity was measured quantitatively using Li-Core infrared detection. The upper panel shows a representative blot. The lower panel shows the statistical analysis. Absolute values measured for isd-1 were set to 100% and values obtained for the other strains were expressed in relation to this. The mean and SD of six Independent experiments is shown. Statistical evaluation was performed using a paired two tailed t-test. P-values of <0.05 were regarded as significant and are indicated by*. *** indicate P-values of <0.0001. (B) Each experiment was controlled by loading a part of the sample used for Western blotting on a second acrylamide gel. SDS gels were strained with Coomassie blue and an apparent non-hb band (130kDa) was chosen as a loading control. A representative gel is shown.

As with *S*. *aureus*, the *isd* operon of *S*. *lugdunensis* allows the acquisition of heme as a source of nutrient iron [10, 11]. The first step of heme acquisition is the binding of hemoglobin (hb) to the NEAT domains of the cell surface-displayed receptors. We therefore investigated whether the increased expression of *isd* improves the binding of human hb to the bacterial cell surface. Strains were grown under iron limiting conditions and incubated with human hb. Subsequently the hb was released by boiling and analysed by quantitative Western blotting using the Li-Core infrared technology (Fig 5). The isd-0 strain was virtually unable to bind hb. Importantly the hb binding capacity of the strains increased according to the *isd* copy number. A strain harbouring 2 copies of *isd* bound almost twice as much hb as the single copy variant. Furthermore, the seven copy variant bound almost three times more hb than the single copy variant and 1.7 times more than the isolate with two copies. Equal loading was confirmed by Coomassie staining of a non-hemoglobin band which showed the same intensity in all strains. The results indicate that the GDA of *isd* might have significant impact on the heme acquisition by *S*. *lugdunensis* HKU09-01 thereby providing an advantage when growing in environments where free iron is strictly limited but hb is available.

### Overexpression of Isd provides a competitive advantage

We investigated whether the amplification of *isd* improved the fitness of the strains when growing in iron limited conditions (RPMI with 10μM EDDHA) in the presence of human hb or heme as a sole source of nutrient iron. In order to compare directly two strains growing together, we decided to perform competitive index (CI) experiments. We labelled strains with IPTG-inducible kanamycin (Kan^R^) or erythromycin (Ery^R^) resistance markers using the integrating plasmid pIPI03 [8]. This allowed cultivation of two strains together in the same flask. Competitive fitness was enumerated by performing viable counts selecting for Ery^R^ and Kan^R^ colonies at the beginning and at the end of experiment.

We found that the RecA deficient strains used in the above-mentioned experiments were not suited for the CI analysis. The mutants grew very poorly in RPMI which was not helped by provision of hb or heme. We suspect that this is due to the *recA* mutation which is associated with decreased growth [8]. In addition, heme possesses a strong oxygenase activity which might cause DNA damage [12] that could not be repaired without RecA. Therefore we decided to use RecA positive strains.

Strain isd-1(R^+^) (HKU::*tetK* Δdup, RecA^+^) was used as a standard and plasmid pIPI03:Ery^R^ was integrated into its chromosome. The strain Isd-0(R^+^) (HKU::*tetK* Δisd RecA^+^) was labelled with pIPI03:Kan^R^. Strain isd-2(R^+^) (HKU::*tetK* RecA^+^) was also labelled with pIPI03:Kan^R^ and copy number variants were selected from isd-2(R^+^) as described above. The *isd* copy number of several strains was determined by qPCR (S5 Fig) and the variants isd-1(R^+^) (V6 in S5 Fig), isd-3(R^+^) (V8 in S5 Fig) and isd-4(R^+^) (V1 in S5 Fig) harbouring 1, 3 and 4 copies, respectively, were chosen.

**Fig 6:**
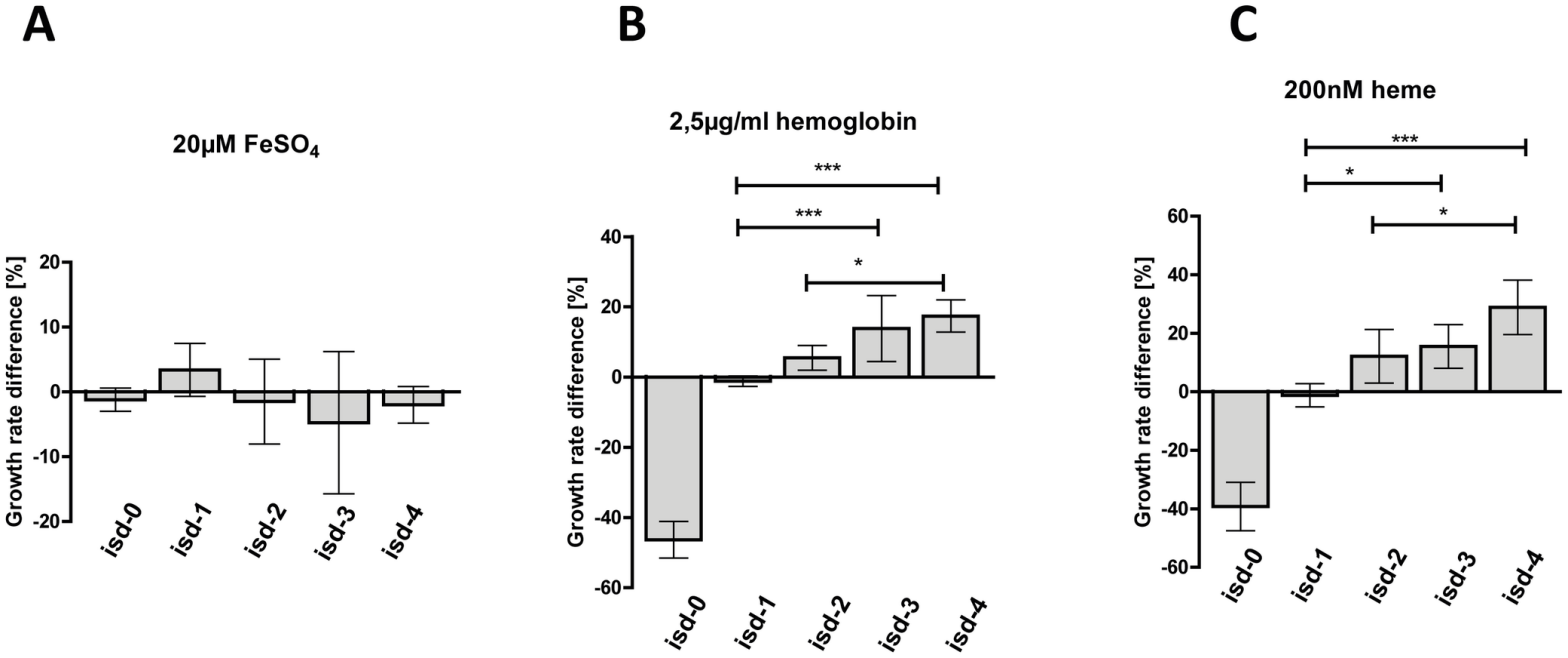
Competitive growth. RPMI (with 10μM EDDHA) cultures were supplemented with (A) 20 μM FeSO_4_, (B) 2,5 μg/ml hemoglobin or (C) 200 nM heme and inoculated with isd-1::pIPI03ery^R^ and one of the *isd* copy number variants: isd-0::pIPI03Kan^R^, isd-1:: pIPI03Kan^R^, isd-2:: pIPI03Kan^R^, isd-3:: pIPI03Kan^R^, isd-4:: pIPI03Kan^R^. All strains used were RecA positive. The CFU of each strain was enumerated by plating on erythromycin and kanamycin containing agar plates. The change in the ratio of the strains was used to calculate the growth rate of each copy number variant in comparison to the single copy control strain. The percentage difference between the growth rate of the isd-1 and copy number variants is shown. The mean and SD of (A) N = 4, (B,C) N = 6 independent experiments is shown. Statistical analysis was performed using One Way Anova followed by Bonferroni’s correction. P-values of <0.05 were regarded as significant and are indicated by*. *** indicate P-values of <0.0001.

The copy number variants (all labelled with Kan^R^) were then grown in direct competition with isd-1::Ery and the relative growth rate (W_isdX_) compared to the single copy control strain was calculated. For clarity, the observed differences between W_isdX_ and W_isd-1_ were expressed (Fig 6A). Alternatively, the individual growth rates of the copy number variants are given in S6 Fig. The use of pIPI03 in CI experiments has been tested extensively and Kan^R^ and Ery^R^ cassettes were not found to influence the outcome [8]. Therefore, the antibiotic markers did not need to be exchanged.

When FeSO_4_ was added as an iron source, none of the copy number variants showed a significant difference compared to the single copy control strain (Fig 6A). This is not surprising, since acquisition of Fe^2+^ is independent of *isd*. Interestingly, we observed a clear but yet not statistically significant trend towards a competitive disadvantage associated with the higher copy number under these conditions. This seems comprehensible since synthesis of additional DNA and protein would be associated with a physiological burden as long as the gene dosage is not associated with a selective advantage (a closer investigation of this phenomenon is described later on).

However, the picture changed dramatically when either hemoglobin or heme was added as a source of nutrient iron (Fig 6B and 6C). Under these conditions, the isd-0 (R^+^) strain was outcompeted by the single copy variant, underlining the importance of *isd* under these conditions. The higher the copy number, the greater the growth advantage the strains possessed over the single copy variant. The highest difference was observed for isd-4 (R^+^) that showed a growth advantage of ~30% over the single copy variant. These results clearly demonstrate that the increased gene dosage of *isd* provides an advantage when competing for hb or heme.

**Fig 7:**
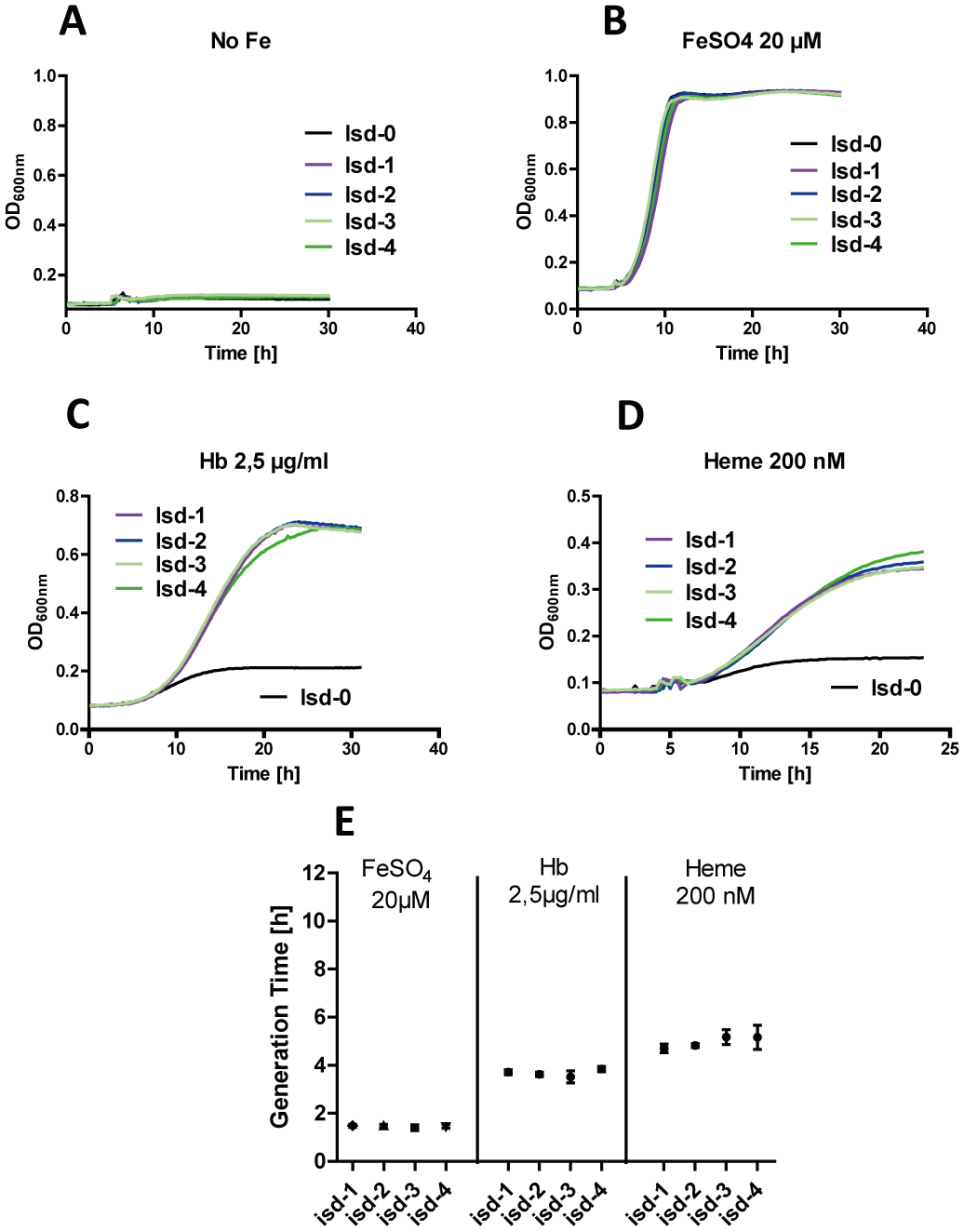
Hemoglobin/heme dependent growth in individual cultures. (A-D) representative growth curves of all copy number variants (RecA positive) grown individually in RPMI (10 μM EDDHA) without additional iron (A) or in the presence of presence of 20 μM FeSO_4_, 2,5 μg/ml hb or 200 nM heme (B-D). The experiment was performed in 48 well microtiter plates using an Epoch2 reader with 300 rpm shaking at 37°C. OD_600nm_ was determined every 15 minutes. (E) Generation times of strains calculated form growth experiments describe in (B-D). Shown is the mean and SD of six independent experiments. Statistical evaluation was performed using One Way Anova followed by Bonferroni’s correction. P-values of <0.05 were regarded as significant and are indicated by*. *** indicate P-values of <0.0001.

Of note, no significant differences in the growth characteristics were observed when strains were grown under iron-restricted conditions in the presence of hb or heme individually. This indicates that without direct competition with other copy number variants, the amplification (up to four copies) confers neither a physiological advantage nor a disadvantage Fig 7. This observation is of interest, since many reports show that GDAs frequently harbour a physiological burden and are rapidly lost from the population when selective pressures are removed. It is suggested that the physiological cost of GDAs is not due to the maintenance of DNA but caused by increased protein expression levels which is energetically costly [13].

In this regard, *isd* is of special interest since the genes are tightly controlled and only expressed under strict iron limitation, which might influence the metabolic cost of the GDA.

We studied the generation times of the copy number variants in TSA (iron rich) and RPMI (iron restricted) that are known to repress and induce *isd* expression, respectively [10]. This allowed us to study the metabolic burdens of the GDA without any positive selection. We found that in a RecA positive background the copy number variation (1–4) copies did not influence the growth rates significantly, although a trend towards slower growth in RPMI was observed for isd-3 and isd-4 (S7 Fig). Therefore we turned to the highest copy number in our selection (isd-7) and repeated the analysis in the corresponding *ΔrecA* background. This time a growth deficit of isd-7 was obvious under iron restricted conditions (RPMI). In contrast no differences between the strains was apparent under iron saturated conditions (TSB) (S7 Fig). Growing the strains in RPMI supplemented with 20 μM FeSO_4_ repressed *isd* expression and eliminated the growth deficit of isd-7 (S7 Fig), confirming that the extensive overexpression of *isd* leads to a growth deficit. This strengthens the hypothesis that a physiological burden of the GDA is inherent. Yet the effects are dependent on protein expression and are buffered under iron-saturated conditions.

### Growth in human serum

Our data indicate that amplification of *isd* provides a significant advantage when strains compete for limited amounts of free hb in their environments. We wondered whether this phenotype is also of importance during infection. However, conventional mouse infection models had very limited chances of success since the Isd system of *S*. *lugdunensis* was shown to be highly human specific and *S*. *lugdunensis* does not tolerate mouse hb as a source of nutrient iron [14]. As an alternative to infection models we decided to investigate the importance of *isd* for growth in human serum. We tested several methods of taking blood and were able to recover serum containing ca. 50 μg/ml hb. However, compared to 2,5 μg/ml used in the previous experiments even 50% diluted serum contained a 10-fold excess of hb. This excess of hb together with the low growth achieved in human serum delayed the Isd dependent effects. Therefore we prolonged our competitive experiments by inoculating consecutive cultures and enumerating the ratios of the strains each day.

**Fig 8:**
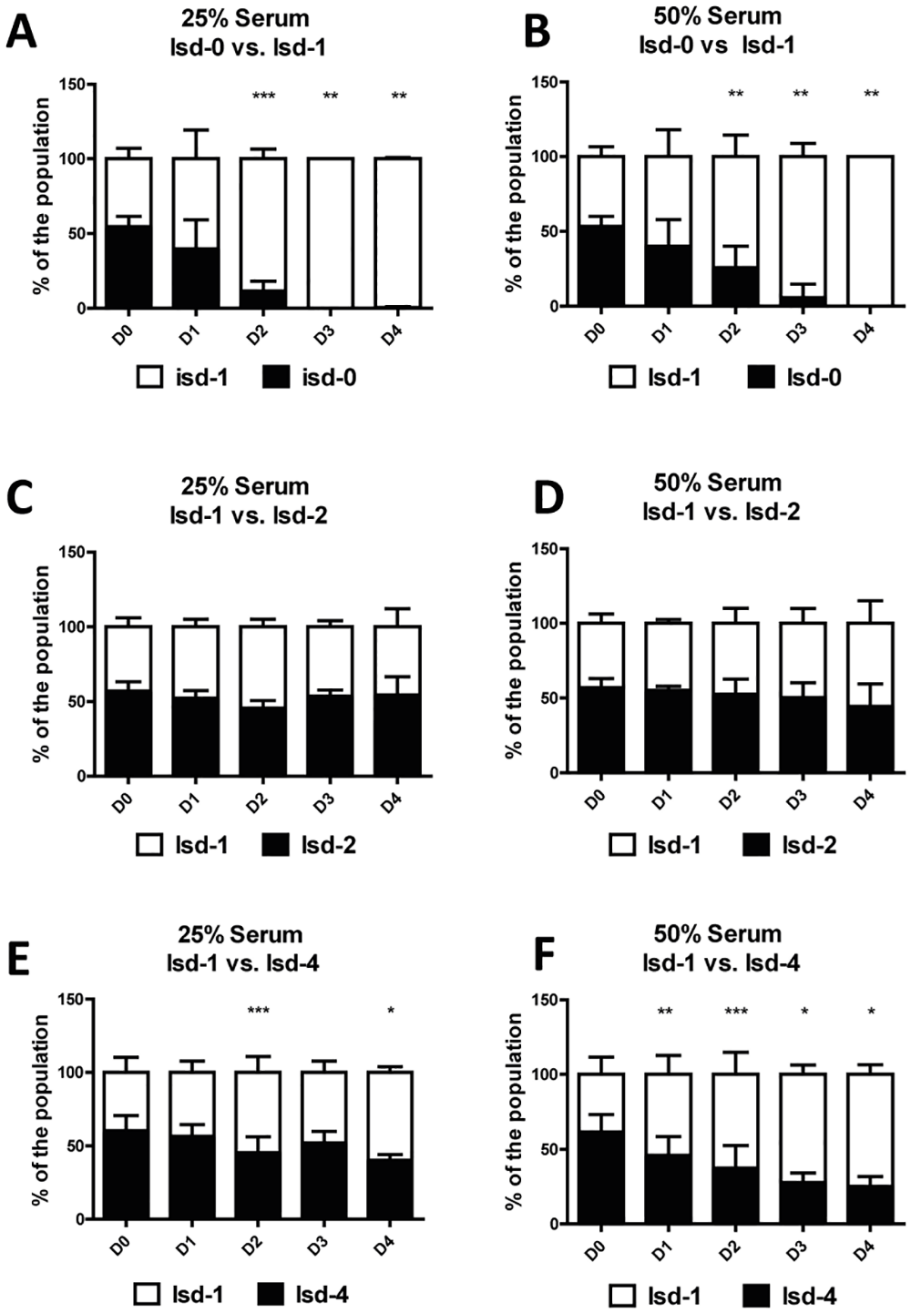
Growth in Human Serum. Human serum was inoculated for competitive growth. (A,B) isd-1::pIPI03ery^R^ vs. isd-0::pIPI03Kan^R^ in 25% and 50% serum (C,D) isd-1::pIPI03ery^R^ vs. isd-2::pIPI03Kan^R^ in 25% and 50% serum (E,F) isd-1::pIPI03ery^R^ vs. isd-4::pIPI03Kan^R^ in 25% and 50% serum. All strains used were RecA positive. After 24 h of growth new serum was inoculated 1:10 with previous sample. The process was repeated up to three times. The CFU of each strain after each cycle of growth was enumerated by plating on erythromycin and kanamycin containing agar plates. The ratio of the two strains in each culture is given. Shown is the mean and SD of six experiments (D0-D2) and three experiments (D3-D4) respectively. For Isd-1 vs Isd-2 mean and SD of four experiments is given. The proportion of the Isd-0, isd-1 and isd-4 after each culture (D1-D4) was compared to the respective proportion at the beginning of the experiment (D0). Statistical evaluation was performed using a paired two tailed t-test. P-values of <0.05 were regarded as significant and are indicated by*. ** indicate P-values < 0,005 and *** indicate P-values of <0.0001.

We found that under both conditions (50% and 25%) of serum the Isd-expressing strain had a competitive advantage. Isd-1 outcompeted Isd-0 stepwise over the consecutive cultures proving Isd to be an essential factor under these conditions (Fig 8A and 8B). However, we found the ratio of isd-1 and isd-2 remained stable over the course of the experiment (Fig 8C and 8D) and we even found that Isd-4 did not possess an advantage over isd-1. In contrast, isd-4 was slowly outcompeted by isd-1 with the effect being more pronounced in 50% serum than in 25% serum (Fig 8E and 8F). This effect is most likely due to the excess of hb, restricting the competition between the strains.

### Hemoglobin binding capacities of various *S*. *lugdunensis* clinical isolates

Our data suggest that the acquisition of heme is of crucial importance to *S*. *lugdunensis*. Therefore, we sought to investigate whether strains of different clonal origins might also differ in their capacity to bind hb to their cell surface. We choose members of all five clonal complexes (CC) described for *S*. *lugdunensis* and found that the strains differed strongly in their capacity to bind hb (S8 Fig). Strains SL09 (CC5), SL27 (CC4) bound a comparable amount of hb to HKU09-01 isd-1. In contrast, SL57 (CC3) bound significantly less hb and most interestingly strain N920143 (CC1) bound as much hb as HKU09.01 isd-4. However, while all of these strains carried the *isd* locus (confirmed by PCR) none of the strains harbored a duplication like HKU09-01. The different hb binding profiles are therefore most likely due to differences in the Isd expression levels or to the presence of additional hb receptors.

## Discussion

Tandem repeat duplications have best been studied in Gram-negative model organisms such as *Escherichia coli* [15] and *Salmonella enterica* [16]. The genetic mechanism for the creation of GDAs involves non-equal homologous recombination mediated by RecA [1]. Such recombination requires regions of homologous DNA which can be provided by ribosomal RNA operons (*rrn*) [16], insertion sequences or transposable elements [17–19]. During the replication of the bacterial chromosome, the two sister chromatids remain in close proximity for a short period of time. The RecA protein can recognize homologous sequences on the sister chromatids and promote recombination, thereby duplicating the region in-between the repetitive sequences. After the primary duplication event, long perfect tandem repeats are generated which allow RecA-dependent amplification and, conversely, the loss of the duplication (segregation) at high frequency. This allows an accordion-like expansion and contraction of the gene array.

GDAs are increasingly recognized to be of fundamental evolutionary importance. Due to the strong impact of the gene dosage on protein biosynthesis, a physiological burden is inherent. Therefore, GDAs are only retained in bacterial populations for as long as the increased protein expression represents a selective advantage. Otherwise, the GDAs will rapidly be lost from the population due to segregation and genetic drift. It was suggested that GDAs drive bacterial evolution by providing increased amounts of important chromosomal loci. This enables the increased acquisition of mutations, deleterious mutations are buffered and positive mutations are retained. Thereby gene function is improved stepwise and even the neo-functionalization of genes is fostered while the original function of the gene is retained on other copies [20], a hypothesis that has recently been strengthened with experimental evidence [21].

However, we lack knowledge about the importance of this mechanism for pathogens during the adaption to the hostile environment created by immune defence mechanisms. Two known systems are *Vibrio cholera* where the *ctxA* and *ctxB* genes encoding the two cholera toxin subunits are duplicated in many clinical isolates [22, 23] and invasive isolates of *Haemophilus influenzae* that carry an amplified *capB* locus where the increased copy number correlates with an increase in invasiveness [24] and a decrease in complement-mediated killing and opsonisation [25, 26]. The GDA of *isd* in *S*. *lugdunensis* HKU09-01 is the first example of an amplification improving bacterial fitness by improving nutrient acquisition under conditions mimicking the nutrient availability in the human body during infection. Iron is an important molecule for the host and the pathogen, since it is required as prosthetic group or cofactor for many enzymes in essential processes such as DNA replication and aerobic respiration [27, 28]. Greater than 90% of the iron in mammals is located intracellularly and is therefore not accessible for extracellular pathogens unless it can be liberated from the cells [29]. Intracellular iron is either engaged as a cofactor or prosthetic group into biochemical reactions or complexed to the tetrapyrrole ring of heme and stored by hemoproteins such as hemoglobin [30]. Extracellular iron is bound by high affinity iron chelating proteins such as lactoferrin and transferrin found in lymph and mucosal secretions or within the serum, respectively. The process of iron sequestration potently inhibits bacterial proliferation and is referred to as “nutritional immunity” [4, 31].

The iron responsive surface determinant locus (*isd*) represents an outstanding characteristic of *S*. *lugdunensis*. The locus has been investigated in detail in *S*. *aureus* and allows the acquisition of heme as a source of nutrient iron. The locus therefore indicates an adaption towards an invasive lifestyle and amongst staphylococcal species it is exclusively present in *S*. *aureus* and *S*. *lugdunensis*. Because of this fact the locus sparked interest in the recent years and its function as a heme acquisition system was confirmed [10, 11, 32–34]. We found Isd to provide an essential advantage during growth of *S*. *lugdunensis* in a human body fluid confirming the importance of the locus even further.

Interestingly the *isd* loci of *S*. *aureus* and *S*. *lugdunensis* are strikingly similar but do also harbour significant differences [5]. A major difference is their chromosomal location. While in *S*. *aureus* the locus is found close to the replication terminus, the locus of *S*. *lugdunensis* is in close proximity to the origin of replication. This is particularly interesting since genes close to the origin are in general regarded to be present at a higher copy number during replication. The duplication of *isd* within the clinical isolate HKU09-01 further underlines the importance of this locus for *S*. *lugdunensis*. The work presented here demonstrates that RecA mediated mechanisms drive the amplification and segregation of the duplicated region in HKU09-01. It was shown that the duplication is intrinsically unstable and subject of amplification events. This happened constantly and without selection resulting in a diverse population after growth in liquid culture. However, the ongoing recombination created lineages with strikingly different phenotypic characteristics. The GDA of *isd* led to an increase of Isd protein expression, thereby allowing a higher amount of hemoglobin to bind to the cell surface. Furthermore, the GDA improved the competitive fitness of the strains resulting in a growth advantage of up to 30% (four copy variant compared to a single copy variant) under iron limitation in the presence of hb or heme. Similar conditions are created in the human body by nutritional immunity. Interestingly, differences between the strains were only apparent when copy number variants competed directly for a limited amount of hb/heme in the environment. This suggests that the ongoing competition between individual lineages of the same clonal origin drives the evolution of the locus in HKU09-01.

Strain HKU09-01 is a clinical isolate, although the type of infection that it caused was not described precisely in the original publication [7]. It was described as “isolated from pus” indicating abscess formation or a similar superficial skin infection. It was shown previously that erythrocytes are present within abscesses making hb an available source of iron at this site [35]. Accordingly, it is tempting to speculate that iron-limitation in the presence of hemoglobin might have selected for the duplication of the *isd* region in HKU09-01.

The Isd system of *S*. *lugdunensis* was shown to be human specific and not to tolerate mouse hb [10, 14]. This fact prevented us from establishing mouse disease models and to challenge our hypothesis *in vivo*. Alternatively, we investigated the importance of Isd for growth in human serum. This method is also problematic since stowage of blood within the veins, puncture of the veins and the associated fall of pressure and finally the manipulation of blood to gain the serum lyses significant amounts of erythrocytes. This leads to a release of hb which is not recycled as it would be in the human body. Drawing and treating blood as gently as possible enabled us to reduce the amount of free hb in serum to ca. 50 μg/ml (100 μg/ml are typical for conventional blood drawing methods). However, these levels of hb are most likely still unphysiologically high. Yet, we found that even under these concentrations isd-1 outcompeted isd-0 confirming the essential nature of Isd. In contrast, amplification of the locus to four lead to a competitive disadvantage in our assays while duplication had no detectable effect. The reason for this is most likely found in the high concentration of hb that limits the competition of the strains for the essential nutrient which is most likely not growth limiting is this assay. Furthermore, the amplification of *isd* might simply result in acquisition of too much heme if an excess of hb is present in the environment. Heme is a potent oxygenase and an overload is known to have toxic side effects [12]. This might explain the growth deficit of isd-4 observed in our assays. *S*. *lugdunensis* does not encode a heme efflux system similar to the *S*. *aureus* HrtAB system. As such it is unclear how *S*. *lugdunensis* can overcome heme-mediated toxicity.

It is suggested that duplications confer an intrinsic physiological burden leading to a growth disadvantage if positive selection is removed. In our experiments we did not observe differences in fitness when the copy number variants were grown in individual cultures in a hb/heme dependent manner suggesting a rather neutral physiological cost under these conditions. However, if the proteins are expressed without any benefit (RPMI without hg/heme), the isd-7 variant displayed a significantly longer doubling time compared to the isd-1 variant. This phenotype was dependent on *isd* expression, strengthening the idea that gene content and expression determines the metabolic burden rather than maintenance of additional DNA. Nevertheless, the metabolic burden of the *isd* amplification seems rather minor and was only clearly measurable in the isolate carrying seven copies of the array. These findings support the idea that GDAs represent double-edged swords, providing an evolutionary advantage only if selective pressure is maintained.

A similar duplication of *isd* cannot occur in *S*. *aureus* due to the different chromosomal context. Not many duplications have so far been reported in any staphylococcal species but GDAs influencing methicillin resistance [36], vancomycin resistance [37], natural competence [38] and WTA biosynthesis [39] were found in *S*. *aureus*. However, these events seem to appear sporadically and are not reported frequently. Whether the amplification of virulence factors is of general importance for staphylococci remains an open question that needs attention in the future. The duplication of *isd* seems to be very specific for the *S*. *lugdunensis* clone HKU09-01. We screened all *S*. *lugdunensis* strains in our collection (57 isolates) and did not detect a similar duplication in any strain, speaking against a frequent occurrence of this particular event. However, our screening process can only detect GDAs with the same recombination point found in HKU09-01. Similar GDAs with different fusion points cannot be detected using this approach. Furthermore, our culture collection contained 37 strains derived from non-infected carriers without invasive disease. In such isolates, a random GDA of *isd* would most likely be lost due to the absence of selective pressures.

GDAs are understood to represent a means for the adaption to environmental conditions, where an increased gene dosage leads to improved fitness. As such, only genes and loci that possess an impaired function or a beneficial but low level side activity will be affected. Overexpression allows bacteria to overcome the problem with the function of the protein and the increase in the DNA template amount allows an increased mutation frequency and thereby an improvement of the protein [16, 40]. Alternatively new genes can develop where the side function becomes dominant [21]. Recently duplications were also found that covered several non-coding RNAs and contributed to the development of novel regulatory functions [41]. Looking at the duplication of *isd* in an evolutionary context might therefore lead to several interesting questions. 1) Is the *isd* operon in *S*. *lugdunensis* not functioning very well in HKU09-01 and might this be the case for other *S*. *lugdunensis* lineages? The presence of *isd* seems unusual for CoNS, so maybe the species acquired the locus only recently and is in a process of development from a skin commensal to a pathogen. The acquisition of iron seems to play a special role for *S*. *lugdunensis* since apart of *isd*, the genes encoding the xenosiderophore uptake system Sst are also duplicated in HKU09-01. Interestingly this duplication is conserved in the clinical isolate N920143 indicating that the species is in the middle of adapting to the use of specific iron sources available in humans. 2) Are there side activities present in the locus that need to be improved? Membrane transport systems seem to be promising candidates. It is easily imaginable that the substrate specificity is not exclusive and the transport of various molecules with different efficiencies might be allowed. GDA might therefore improve the import/export of molecules other than heme and siderophores (*sirABC*). However, such questions remain speculation since they are very difficult to address with the experimental systems available up to date, especially if any putative side function of the proteins of interest are not understood.

GDAs involving virulence or fitness factors of pathogens are rarely described and investigated. It is unclear whether this is due to a rare occurrence or to the fact that limited attention is paid to their presence. However, studying GDAs holds an immense potential for infection research. GDAs are unstable and would be lost without selection. As such, any GDA identified was most likely beneficial under the natural growth conditions of the isolate. Gene products affected by GDAs might therefore represent attractive targets for antibiotic intervention with novel drugs to support the immune functions and to prevent bacterial adaption.

## Materials and Methods

If not stated otherwise, all reagents were obtained from Sigma Aldrich

### Bacterial strains and growth conditions

All bacterial strains are listed in Table 1. If not specified otherwise, *S*. *lugdunensis* strains were grown at 37°C in Tryptic Soy Broth (TSB) or on Tryptic Soy Agar (TSA) with shaking at 200 rpm for liquid cultures. *E*. *coli* strains were grown at 37°C Luria Bertani Broth (LB) or on Luria Bertani Agar (LA) (Difco) with shaking at 200 rpm for liquid cultures.

**Table 1:** Bacterial strains and plasmids used in this study.

Strain/Plasmid	Isd copy number designation	description	Source
**Bacterial strains**			
*S*. *lugdunensis* N920143		Sequenced WT strain Clonal complex 1	[5]
*S*. *lugdunensis* HKU09-01		Sequenced WT strain Clonal complex 1	[7]
*S*. *lugdunensis* HKU::*tetK* Δisd *ΔrecA*	isd-0	*tetK* integrated, no duplication of the *isd* region, deletion of the entire *isd* locus, deletion of *recA*	This study
*S*. *lugdunensis* HKU:: *tetK* Δdup *ΔrecA*	isd-1	*tetK* integrated, no duplication of the *isd* region, deletion of *recA*	This study
*S*. *lugdunensis* HKU:: *tetK ΔrecA*	isd-2	*tetK* integrated, duplication of the *isd* locus, deletion of *recA*	This study
*S*. *lugdunensis* HKU:: *tetK* Y1 *ΔrecA*	isd-5	*tetK* integrated, amplification of the *isd* locus (5 copies), deletion of *recA*	This study
*S*. *lugdunensis* HKU:: *tetK* X1 *ΔrecA*	isd-7	*tetK* integrated, amplification of the *isd* locus (7 copies), deletion of *recA*	This study
*S*. *lugdunensis* HKU:: *tetK* Z1 *ΔrecA*	isd-5	*tetK* integrated, amplification of the *isd* locus (5 copies), deletion of *recA*	This study
*S*. *lugdunensis* HKU:: *tetK* W1 *ΔrecA*	isd-7	*tetK* integrated, amplification of the *isd* locus (7 copies), deletion of *recA*	This study
*S*. *lugdunensis* HKU:: *tetK* W2 *ΔrecA*	isd-4	*tetK* integrated, amplification of the *isd* locus (4 copies), deletion of *recA*	This study
*S*. *lugdunensis* HKU:: *tetK* Δisd *recA*^+^	isd-0 (R^+^)	*tetK* integrated, no duplication of the *isd* region, deletion of the entire *isd* locus, deletion of *recA* positive	This study
*S*. *lugdunensis* HKU:: *tetK* Δdup *recA*^+^	isd-1 (R^+^)	*tetK* integrated, no duplication of the *isd* region, *recA* positive	This study
*S*. *lugdunensis* HKU:: *tetK recA*^+^	isd-2 (R^+^)	*tetK* integrated, duplication of the *isd* locus, *recA* positive	This study
*S*. *lugdunensis* HKU:: *tetK* V1 *recA*^+^	isd-4 (R^+^)	*tetK* integrated, amplification of the *isd* locus (4 copies), *recA* positive	This study
*S*. *lugdunensis* HKU:: *tetK* V2 *recA*^+^	isd-3 (R^+^)	*tetK* integrated, amplification of the *isd* locus (3 copies), *recA* positive	This study
*S*. *lugdunensis* HKU:: *tetK* V3 *recA*^+^	isd-3 (R^+^)	*tetK* integrated, amplification of the *isd* locus (3 copies), *recA* positive	This study
*S*. *lugdunensis* HKU:: *tetK* V4 *recA*^+^	isd-2 (R^+^)	*tetK* integrated, amplification of the *isd* locus (2 copies), *recA* positive	This study
*S*. *lugdunensis* HKU:: *tetK* V5 *recA*^+^	isd-3 (R^+^)	*tetK* integrated, amplification of the *isd* locus (3 copies), *recA* positive	This study
*S*. *lugdunensis* HKU:: *tetK* V6 *recA*^+^	isd-1 (R^+^)	*tetK* integrated, amplification of the *isd* locus (1 copy), *recA* positive	This study
*S*. *lugdunensis* HKU:: *tetK* V7 *recA*^+^	isd-3 (R^+^)	*tetK* integrated, amplification of the *isd* locus (3 copies), *recA* positive	This study
*S*. *lugdunensis* HKU:: *tetK* V8 *recA*^+^	isd-3 (R^+^)	*tetK* integrated, amplification of the *isd* locus (3 copies), *recA* positive	This study
*S*. *lugdunensis* SL09		Clonal complex 5	[46]
*S*. *lugdunensis* SL27		Clonal complex 4	[46]
*S*. *lugdunensis* SL57		Clonal complex 3	[46]
*S*. *lugdunensis* SL71		Clonal complex 2	[46]
**Plasmids**			
pT181		Staphylococcal plasmid conferring Tc^R^	[47]
pIMAY		Thermosensitive vector for allelic exchange	[48]
pIMAY:*tetK*		Fragment to insert the *tetK* into the duplicated region of HKU09-01	This study
pIMAY:Δ*recA*		*recA* deletion plasmid	[8]
pIMAY:Δisd		Plasmid for the deletion of the entire isd locus	[10]
pIPI03		Site-specific integration vector for HKU09-01	[8]
pIPI03:*ery*		pIPI03 carrying IPTG-inducible *ermAM* genes from pIMC*ery* within the MCS	[8]
pIPI03:*kan*		pIPI03 carrying the IPTG-inducible *aphA3* gene from pIMC*kan* within the MCS	[8]

### Isolation of DNA

The Wizard *Plus* SV Miniprep kit (Promega) was used for isolation of plasmid DNA according to the manufacturer’s protocol. The PureElute Bacterial Genomic kit (Edge Biosystems) was used for the isolation of chromosomal DNA from *S*. *lugdunensis* (10 ml culture) with an additional incubation step with lysostaphin (Ambi Products LLC10 μl of a10 mg/ml stock) and mutanolysin 10 μl (5000 U/ml) at 37°C for 20 min prior to lysis.

### Construction of the pIMAY:tetK integration cassette

Two 500 bp fragments, one upstream and one downstream of the chosen insertion site in the *S*. *lugdunensis* chromosome were PCR-amplified using the primer combinations tetK-A / tetK-B and tetK-C / tetK-D, respectively (Table 2). The two fragments were fused together using “splicing by overlapping extension” (SOE) PCR resulting in a single 1 kb fragment. A novel HindIII restriction site (inserted in primers B and C) was thereby introduced between the fragments AB and CD. The recombinant fragment was cloned into the SacI / KpnI sites of the multiple cloning site of pBluescript. The 2.3 kb *tetK* fragment of pT181 was PCR-amplified (primers tetK-F. / tetK-R.) harbouring a HindIII site at both ends. The fragment was subsequently cloned into HindIII-cleaved pBluscript:AB/CD. The resulting integration cassette AB- *tetK*–CD was excised from pBluescript using the restriction endonucleases NotI and AatLI and cloned into pIMAY treated with the same endonucleases.

**Table 2:** Oligonucleotides used in this study.

Name	5’-3’ Sequence	Purpose
dupl.-F	GGATAAGTACCCAAATACTAACACTGTTGAC	Screening primer directed against the boundaries of the duplication
dupl.-R	TACAATGCTTAACTGTAAAATAATTGGAATATACC	Screening primer directed against the boundaries of the duplication
Prb-F.	AATATCTTCCACATAACAACCTCC	Amplification of the probe for Southern blotting
Prb-R.	TTTTAGAAGGTGAAGGTGCC	Amplification of the probe for Southern blotting
tetK-A	ATTTTTTCAGAGCTCATGGCTTTCGTTAAGATG	Cloning of the *tetK* integration cassette
tetK -B	AAGCTTATTGAAAACCCCGTTGACACTCATC	Cloning of the *tetK* integration cassette
tetK -C	ACGGGGTTTTCAATAAGCTTATGTTTTGTTAGTGTTATGTG	Cloning of the *tetK* integration cassette
tetK -D	ATATAACGGGTACCTTTGAAACACCATTGC	Cloning of the *tetK* integration cassette
tetK -F	GTCAACGGGGTTTTCAATGGGGAAAGCTTCACAGAA	Amplification of *tetK* from pT181
tetK -R	CATAACACTAACAAAACATCGCTGTTAAAGCTTTTTTATTAC	Amplification of *tetK* from pT181
IsdJ-F	CGGCTTCAATTATCGTTGGT	qPCR determination of *isd* copy number
IsdJ-R	TGTGGCAGCTTGATTTTGAG	Determination of *isd* copy number
Ori-F	TCCCGTAACTGTTATCGTCAAA	qPCR determination of *isd* copy number
Ori-R	TCGAGCAACTTATCCACACAA	qPCR determination of *isd* copy number

### Allelic exchange in *S*. *lugdunensis*

Transformation of *S*. *lugdunensis* and allelic exchange was performed to integrate *tetK* into the chromosome of HKU09-01 and to create the *recA* deletion mutation in several strains. The procedure is described elsewhere [42].

### Southern blotting

Digoxigenin (Dig) labelled molecular weight markers were supplied by Roche. Genomic DNA (3 μg) was cleaved for 18 h using ApaLI (Fermentas) and separated by electrophoresis through a 0.8% agarose gel (40 V for 22 h). The DNA was denatured by submerging the gel for 45 min in DN buffer (1.5 M NaCl, 0.5 M NaOH). The gel was rinsed with ddH_2_O, and neutralized for 45 min in NE buffer (1.5 M NaCl, 1 M Tris-HCl pH 7.4). The DNA fragments were transferred to a nylon membrane (Roche) using overnight capillary transfer with 20 x SCC buffer (3 M NaCl, 0.3 M Na_3_Citrate). After transfer the DNA was heat-fixed (80°C for 2 h) to the membrane. Non-specific binding was blocked for 4 h at 70°C using 30 ml of BL buffer (5 x SCC buffer, 0.1% N-lauroylsarcosine, 0.02% SDS, 1 x blocking reagent (Roche). The 600 bp DNA probe was amplified by PCR using primers Prb-F / Prb-R and labelled with Digoxygenin (Roche) according to the supplier’s recommendation. The probe was denatured by boiling for 10 min. Hybridization was carried out overnight at 70°C using 30 ml BL buffer containing 15 ng/ml probe. The membrane was washed twice for 10 min with 50 ml W1 (5 x SSC, 0.1% SCC) at room temperature and twice for 20 min with 50 ml W2 (0.5 x SCC, 0.1% SDS) at 68°C. Additional washing was performed at room temperature with Buffer-1 (0.1 M maleic acid, 0.15 M NaCl, pH 7.5). The membrane was washed for 5 min with 50 ml Buffer-1 (with 0.3% Tween 20) and 30 min with 50 ml Buffer-1 (containing 1 x blocking reagent). Anti-Dig-AP Fab fragments (Roche) were diluted 1: 10000 in Buffer-1 (containing 1 x blocking Reagent) and 40 ml were added to the membrane and incubated for 30 min at room temperature. The membrane was washed twice with 50 ml Buffer-1 (with 0.3% Tween 20). The membrane was equilibrated for 5 min in 30 ml EB (100 mM Tris-HCl, 100 mM NaCl, pH 9.5)., CSPD substrate solution (Roche) was diluted 1: 100 in EB and 5 ml were added to the membrane and incubated for 5 min. The solution was poured off the membrane and the membrane was sealed in a hybridization bag (Roche). Immunoreactive bands were detected with the “ImageQuant Las 4000” system and the corresponding “ImageQuant TL” Software.

### *S*. *lugdunensis* cell fractionation

Fractionation was carried out as described earlier [43] with minor modifications. Cells were grown in RPMI to stationary phase and washed once with buffer WB (10 mM Tris-HCl pH 7, 10 mM MgCl). A 1 ml aliquot of cells adjusted to an OD_578_ = 5 was centrifuged (18000 x g) and resuspended in 100 μl buffer DB (10 mM Tris-HCl pH 7, 10 mM MgCl, 500 mM sucrose, 0.3 mg/ml lysostaphin, 250 U/ml mutanolysin, 30 μl protease inhibitor cocktail (Roche– 1 complete mini tablet dissolved in 200 μl H_2_O), 1 mM phenyl-methanesulfonylfluoride (PSMF). The digestion of the cell wall was carried out at 37°C for 1 h followed by centrifugation (3000 x g for20 min at 4°C). The supernatant was designated the cell wall fraction. The pellet containing the protoplasts was washed with 1 ml WB (with 500 mM sucrose) and centrifuged again as above. The protoplasts were resuspended in 200 μl buffer LB (100 mM Tris-HCl pH 7, 10 mM MgCl, 100 mM NaCl, 10 μg/ml DnaseI, 100 μg/ml RnaseA). The suspension was frozen and thawed three times to ensure protoplast lysis and centrifuged for 30 min (18,000 x g at 4°C). The pellet (designated the membrane fraction) was washed with 1 ml of buffer LB and resuspended in 100 μl TE buffer (100 mM Tris-HCl pH 8, 1 mM EDTA). 5–15 μl of the fractions were used for analysis by SDS-PAGE and Western immunoblotting using standard procedures and antibodies against Isd proteins described elsewhere [10]. DYLight 800 (Sigma) labelled secondary antibodies were used and fluorescence intensity was quantified using the “Odyssey ClX” Infra-red technology.

### Whole cell immunoblotting

Cells were grown to stationary phase in RPMI with 1% casamino acids (CA, Difco). Cells were harvested, washed once with PBS and adjusted to the desired optical densities. The cell suspension (5 μl) was applied to a nitrocellulose membrane (Roche) and allowed to air dry. The membrane was subsequently blocked for 1 h with 10% (w/v) milk powder in TS buffer (10 mM Tris-HCl, 150 mM NaCl, pH 7.4). Membranes were washed 3 times for 10 min with TS buffer and then stained with primary antibodies [10] for 1 h in 10% (w/v) milk powder in TS buffer. Membranes were washed 3 times with TS buffer and stained with goat anti-rabbit DYLight 800 (Sigma). The secondary antibodies were applied in 10% (w/v) milk powder in TS buffer. Finally, the membranes were washed 3 times with TS buffer and fluorescence intensity was quantified using the “Odyssey ClX” Infra-red technology. Only the dots corresponding to the cell concentration of OD_600_ = 0,5 and of OD_600_ = 1 were quantified for IsdC and IsdB, respectively.

### Quantitative PCR to determine the *isd* copy number

Primers used for the experiments are summarized in Table 2. Primers OriF/R amplified a 100 bp fragment at the origin of the chromosome. The amplification of *ori* served as a standard for these experiments since there is only one copy per chromosome. Primers IsdJ F/R amplified a 100 bp internal fragment of *isdJ*. Thus the relative template amount of *isdJ* compared to *ori* could be determined, allowing an estimation of the copy number of *isdJ*.

Chromosomal DNA of N920143 was isolated and 10-fold dilutions (10^−2^ to 10^−6^) were used to generate the standard curves for *ori* and *isdJ*. Chromosomal DNA of the sample strains was isolated and the 10^−4^ dilution was used as template. Each reaction was carried out in a 20 μl volume containing 2 μl template, 150 nM of primer F and R and 10 μl Power SYBR Green PCR Master Mix (Applied Biosystems). Each reaction was performed in duplicate.

The RT-PCR Cycler “StepOnePlus” with the appropriate equipment (96 well plates, adhesive covers) was used for the experiments (Applied Biosystems). Cycling was performed as follows: 2 min at 50°C followed by 10 min at 95°C followed by 40 cycles of 15 s at 95°C and 1 min at 60°C. A melt curve analysis was performed, confirming that only a specific product was generated by the primers used. StepOne software Version 2.1 was used for data analysis.

### PCR colony screen to determine the stability of the duplication in HKU09-01

Single colonies of HKU09-01 wild-type or the isogenic Δ*recA* mutant were used to inoculate TSB cultures. The cultures were grown at 37°C with agitation to stationary phase and dilutions were plated on TSA and incubated overnight at 37°C. 22 colonies were screened by colony PCR for the presence of the duplication using the primers dupl-F and dupl-R (Table 2). A single colony was resuspended in 20 μl TE Buffer (10 mM Tris-HCl, 1 mM EDTA, pH 8), boiled for 10 min and centrifuged (5 min at 10000 x g). 0.5 μl of the supernatant was used for a 25 μl PCR reaction using Phyre hot start polymerase (Finzymes). All clones that did not allow the amplification of a PCR product were screened for a second time to ensure loss of the duplication. Only clones that did not allow the amplification of a product in both PCR screens were regarded as strains without the duplication.

### Hemoglobin binding assay

Bacterial strains were grown in RPMI with 1% CA containing 0.5 mM 2,2’-bipyridyl. Cells were harvested by centrifugation, washed with PBS and adjusted to an OD_600_ = 2. Human hemoglobin was isolated from healthy volunteers as describe elsewhere [44] and added to a final concentration of 10 μg/ml to a 1 ml cell suspension. The mixture was incubated for 30 min at 37°C with mild agitation (300 rpm). Cells were harvested (10 min, 3000 x g, 4°C) and washed thrice with ice-cold PBS. The pellet was resuspended in 50 μl buffer (0,5 M Tis-HCl, 4% SDS pH) and boiled for 5 min to release surface bound hb. After centrifugation (5 min, 18000 x g, 4°) 20 μl aliquots of the supernatant were loaded on two 15% polyacrylamide gels. One of the gels was stained using Coomassie Brilliant Blue to provide a loading control. The second gel was blotted onto a PVDF membrane and hb was detected using rabbit anti-human hb antibodies (Sigma) followed be goat anti-rabbit IRDye-800 (Li-Core). Hb was quantified using the “Odyssey ClX” Infra-Red technology. The hb specific fluorescent signal received for isd-1 was regarded as 100% for each experiment. Signals received for the copy number variants were expressed in relation to isd-1.

### Competitive growth

Bacteria were grown overnight in TSB. Cells were harvested by centrifugation, washed with PBS and adjusted to an OD_600_ = 1. The same volumes of strains labelled with and pIPI03:Ery^R^ and pIPI03:Kan^R^ were mixed and 10 μl were used to inoculate 2 ml of iron-limited test medium (RPMI 1% CA containing 10 μM EDDHA and either 20 μM FeSO_4_, 2,5 μg/ml hb or 200 nM heme (bovine hemin, Sigma). The cultures were incubated for 24 h (37°C at 300 rpm).The absolute numbers of the bacteria of the each strain were enumerated at the beginning and the end of the experiment by performing viable counts on both TSA with 50 μg/ml kanamycin and TSA with10 μg/ml erythromycin. Plating and counting of the CFU was performed using the spiral plating device “EDDY-Jet 2” and the corresponding CFU counting appliances (I&L Biosystems).

All *isd* copy number variants were competed with the single copy control strain HKU::*tetK* Δdup, RecA^+^. The growth rate of this isd-1 control strain in each competition is 1 and the relative growth rate W_var_ of each copy number variant was calculated as follows W_var_ = [ln[N_var(t2)_ x d/N_var(t1)_]] / [ln[(1-N_var(t2)_) x d/ (1-N_var(t1)_)]]. *N*_var(*t*1)_ and *N*_var(*t*2)_ are the proportions of the mutant in the population at the start (*t*_1_) and the end (*t*_2_) of the competition, respectively, and *d* represents the growth of the entire population over the course of the competition (OD_600_ at *t*_2_ / OD_600_ at *t*_1_).The formula was adapted from [45].

### Growth in individual cultures

Bacteria were grown overnight in TSB. Cells were harvested by centrifugation, washed with PBS (for growth in RPMI/TSB) or RPMI with 10 μM EDDHA (for hb/heme dependent growth), adjusted to an OD_600_ = 1 and 2,5 μl were used to inoculate 0,5 ml of the appropriate test medium in individual wells of a 48 well microtiter plate (NUNC). Bacterial growth was monitored for 24–48 h using an Epoch2 reader (300 rpm shaking at 37°C). The OD_600nm_ was determined every 15 minutes. Bacterial generation times during exponential phase were calculated using the following formula $G=\frac{t1-t0}{\begin{array}{c} \left(3,3*\text{log}\left(\frac{b}{B}\right)\right) \end{array}}$ (G- generation time, t_0_- start of exponential phase, t_1_- end of exponential phase, b- OD_600**t1**_, B-OD_600**t0**_.

### Growth in human serum

Human blood was drawn without blood stowage and without the use of vacuum contain tubes. The blood was incubated on ice for 30 min to allow coagulation. After centrifugation (20 min, 1500 g, 4°C) the serum was collected and the amount of free hb was measured using a human hb ELISA kit (Abcam).

Bacterial strains were grown and washed as described above (competitive growth). 2 μl of the OD1 mixtures were used to inoculate 200 μl serum (diluted with PBS 10 μM EDDHA) and grown for 24 h (37°C, 300 rpm) in 15 ml snap cap tubes. After 24 h fresh serum was inoculated 1:10 with the previous sample and incubated again for 24 h. This process was repeated 2 times. Bacterial numbers were enumerated after each incubation by performing viable counts on both TSA with 50 μg/ml kanamycin and TSA with10 μg/ml erythromycin. Plating and counting of the CFU was performed using the spiral plating device “EDDY-Jet 2” and the corresponding CFU counting appliances (I&L Biosystems).

### Ethics statement

Human hemoglobin and human serum was isolated from venous blood of healthy volunteers. The taking of human blood was approved by the Institutional Review Board for Human Subjects at the University of Tübingen “Ethik-Kommission an der Medizinischen Fakultät Tübingen”. Project number 014/2014BO2. Volunteers were informed about risks of the intervention, the aim of the research and randomization of patient data. Written consent was obtained from all volunteers.

### Statistical analysis

Statistical analysis was performed by using GraphPad Prism (GraphPad Software, Inc., La Jolla, USA; version 5.04). Statistically significant differences were calculated by using statistical methods as indicated.

## Supporting Information

S1 Fig Schematic diagram of the duplication in HKU09-01. Diagram of the duplicated region in HKU09-01. The upper part shows the gene organization of the single locus in N920143. Genes upstream and downstream of the *isd* locus with 19 nucleotide homology and are shown in black and white, respectively. The in-frame fusion gene created by the duplication is shown in black/white. The duplicated region encodes the complete *isd* operon and the *sirABC* transporter. Gene IDs are given.
(TIF)

S2 Fig *tetK* insertion site. (A) Schematic diagram of the *tetK* insertion site. *tetk* was inserted at the 3’ end of the duplicated region between the coding sequences SLGD_00110 and SLGD_00111. The *tetK* integration cassette on the thermosensitive plasmid contains *tetK* flanked by regions upstream (AB) and downstream (CD) of the chosen insertion point. Insertion and excision of the thermosensitive plasmid allowed the exchange of plasmid and chromosomal segments and thereby the isolation of HKU::*tetK* carrying the *tetK* gene in the chromosome. (B) Schematic diagram of the restriction fragments created by *tetK* insertion. ApaLI restriction sites are indicated by vertical arrows. The binding site of the DIG-labelled probe is indicated by the black dash. Predicted sizes of the fragments recognized by the probe are indicated. (C) Results of the Southern blot. Chromosomal was digested with ApaLI and separated by electrophoresis. The DNA fragments were subsequently denatured, blotted onto a nylon membrane and hybridized with the DIG-labelled probe. Hybridization was detected using anti-DIG Fab fragments conjugated to alkaline phosphatase.
(TIF)

S3 Fig Organization of the *isd* loci of strains with different *isd* copy numbers. Schematic diagrams of the chromosomal *isd*-encoding regions in the different strains are shown. Brackets indicate the amplified region in isd—7.
(TIF)

S4 Fig IsdB surface expression. Strains were grown in RPMI, adjusted to OD_578_ = 5 and the cell wall was digested with lysostaphin and mutanolysin in the presence of 500 mM sucrose to stabilize the protoplasts. Cell wall and membrane fractions were separated by SDS-PAGE and blotted onto a PVDF membrane. Isd proteins were detected using specific rabbit serum followed by goat anti-rabbit IgG conjugated to HRP. IsdB was detected in the cell wall fraction. The experiment was repeated three times. A representative blot is shown.
(TIF)

S5 Fig qPCR to determine the *isd* copy number in RecA^+^ strains. Known concentrations of N920143 DNA (one copy of *isd*) were used to create the standard curves for *isdJ* and *ori*. Relative amounts of template *ori* and *isdJ* for each strain were measured. The value for *ori* was set to 1 and the template amount of *isdJ* was expressed in relation to this value, thereby giving the copy number of *isdJ* in the chromosome of each strain. The mean and SD of three experiments is shown.
(TIF)

S6 Fig Growth rates of isd copy number variants in relation to isd-1. RPMI (with 10μM EDDHA) cultures were supplemented with (A) 20 μM FeSO_4_, (B) 2,5 μg/ml hemoglobin or (C) 200 nM heme and inoculated with isd-1::pIPI03ery^R^ and one of the *isd* copy number variants: isd-0::pIPI03Kan^R^, isd-1:: pIPI03Kan^R^, isd-2:: pIPI03Kan^R^, isd-3:: pIPI03Kan^R^, isd-4:: pIPI03Kan^R^. All strains used were RecA positive. The CFU of each strain was enumerated by plating on erythromycin and kanamycin containing agar plates. The change in the ratio of the strains was used to calculate the growth rate of each copy number variant in comparison to the single copy control strain (W_isd-1_ was set to 1 for each experiment). The mean and SD of (A) N = 4, (B,C) N = 6 independent experiments is shown. Statistical analysis was performed using One Way Anova followed by Bonferroni’s correction. P-values of <0.05 were regarded as significant and are indicated by*. *** indicate P-values of <0.0001.
(TIF)

S7 Fig Generation times in TSA and RPMI. (A) Generation times of RecA positive copy number variants grown in TSB and RPMI. (B) Generation times of RecA negative copy number variant grown in TSB, RPMI and RPMI supplemented with 20 μM FeSO_4_. Growth assays were performed in 48 well microtiter plates using an Epoch2 reader with 300 rpm shaking at 37°C. OD_600nm_ was determined every 15 minutes. Bacterial generation times were calculated. Shown is the mean and SD of four independent experiments (A) and five independent experiments (B), respectively. Statistical evaluation was performed using One Way Anova followed by Bonferroni’s correction. P-values of <0.05 were regarded as significant and are indicated by*. *** indicate P-values of <0.0001. (C) Whole cell immunoblot: Overnight cultures isd-1 *(ΔrecA*) were grown overnight in RPMI (with and without 20 μM FeSO_4_) adjusted to an OD_578_ = 1 and doubling dilutions were spotted on the membrane. IsdC protein was detected with specific rabbit serum followed by goat anti-rabbit IgG DYLight 800. Fluorescence intensity was measured using Li-Core infrared detection.
(TIF)

S8 Fig Hb binding capacity of various *S*. *lugdunensis* strains. *S*. *lugdunensis* strains (all RecA positive) were grown in RPMI with 0,5mM bipiridyl, adjusted to OD_578_ = 2 and incubated with 10 μg/ml human hemoglobin (hb). After washing cell-surface bound hg was released by boiling and supernatants were separated by SDS-PAGE. Proteins were blotted onto a PVDF membrane. Human hb was detected using specific rabbit serum followed by goat anti-rabbit IgG DYLight 800. Fluorescence intensity was measured quantitatively using Li-Core infrared detection. Absolute values measured for HKU09-01 isd-1 were set to 100% and values obtained for the other strains were expressed in relation to this. The mean and SD of four independent experiments is shown. Strains binding significantly more or significantly less hb than HKU09-01 isd-1 are indicated. Statistical evaluation was performed using a paired two tailed t-test. P-values of <0.05 were regarded as significant and are indicated by*. ** indicate P-values of <0.001.
(TIF)
