# Correction: Competing for Iron: Duplication and Amplification of the *isd* Locus in *Staphylococcus lugdunensis* HKU09-01 Provides a Competitive Advantage to Overcome Nutritional Limitation

**DOI:** 10.1371/journal.pgen.1007564

**Published:** 2018-07-27

**Authors:** Simon Heilbronner, Ian R. Monk, Jeremy R. Brozyna, David E. Heinrichs, Eric P. Skaar, Andreas Peschel, Timothy J. Foster

There is an error in the Funding statement. The correct statement should read as follows:

“We acknowledge the support of the Irish Research Council for Science, Engineering and Technology for an Embark scholarship (RS2000192) to SH (http://www.research.ie/). We acknowledge the support of the Science Foundation Ireland for a Programme Investigator grant (08/IN.1/B1854) to TJF (http://www.sfi.ie/). We acknowledge the support of the German Academic exchange (DAAD) for a stipend for the “retrieval of German Scientist working abroad” (50722682) to SH (https://www.daad.de/en/). We acknowledge the support of the Research Executive Agency to SH (https://erc.europa.eu/). This project has received funding from the European Union’s Horizon 2020 research and innovation programme under the Marie Sklodowska-Curie grant agreement No “GA 655978”. Work in the laboratory of AP was supported by the SFB766 grant from the German Research Council (http://www.dfg.de/en/). Work in the laboratory of EPS is supported by NIH R01 AI069233 and R01 AI073843 (http://grants.nih.gov/grants/oer.htm) and Department of Veterans Affairs Merit Award INFB-024-13F (http://www.research.va.gov/default.cfm). Work in the laboratory of DEH is supported by Canadian Institutes of Health Research grant MOP-38002 (http://www.cihr-irsc.gc.ca/e/193.html). We acknowledge support by "Deutsche Forschungsgemeinschaft" and the "Open Access Publishing Fund" of the University of Tübingen. The funders had no role in study design, data collection and analysis, decision to publish, or preparation of the manuscript.”
