# Supplementary figures and images for: Competing for Iron: Duplication and Amplification of the isd Locus in Staphylococcus lugdunensis HKU09-01 Provides a Competitive Advantage to Overcome Nutritional Limitation
Source: PLoS Genet. 2016 Aug 30;12(8):e1006246. doi: 10.1371/journal.pgen.1006246 (PMC5004866; doi:10.1371/journal.pgen.1006246)

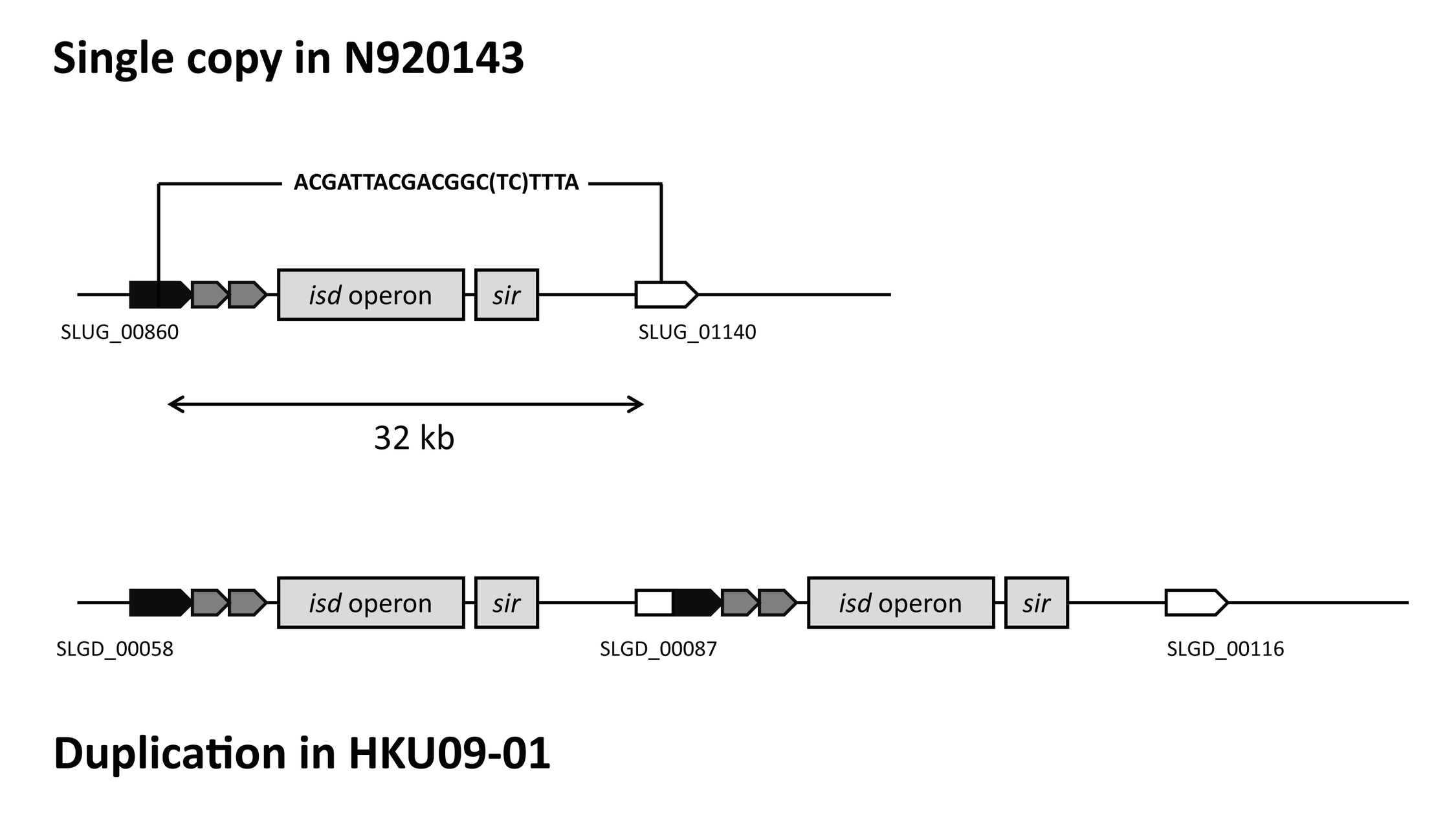

Supplement: S1 Fig — Diagram of the duplicated region in HKU09-01. The upper part shows the gene organization of the single locus in N920143. Genes upstream and downstream of the isd locus with 19 nucleotide homology and are shown in black and white, respectively. The in-frame fusion gene created by the duplication is shown in black/white. The duplicated region encodes the complete isd operon and the sirABC transporter. Gene IDs are given. (TIF) [file pgen.1006246.s001.tif]

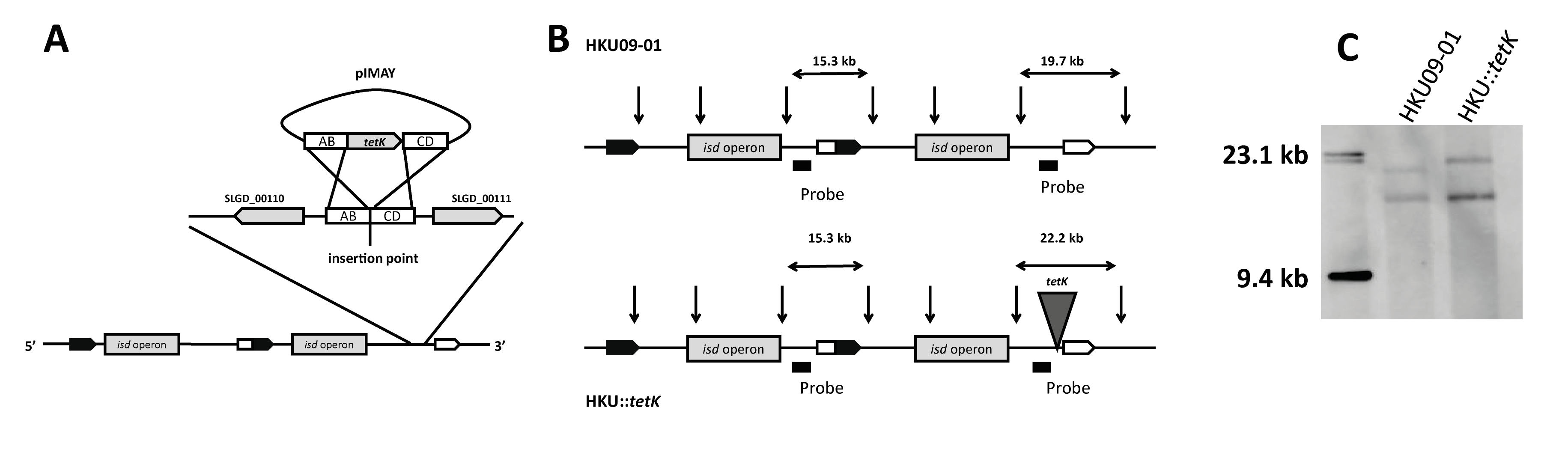

Supplement: S2 Fig — (A) Schematic diagram of the tetK insertion site. tetk was inserted at the 3’ end of the duplicated region between the coding sequences SLGD_00110 and SLGD_00111. The tetK integration cassette on the thermosensitive plasmid contains tetK flanked by regions upstream (AB) and downstream (CD) of the chosen insertion point. Insertion and excision of the thermosensitive plasmid allowed the exchange of plasmid and chromosomal segments and thereby the isolation of HKU::tetK carrying the tetK gene in the chromosome. (B) Schematic diagram of the restriction fragments created by tetK insertion. ApaLI restriction sites are indicated by vertical arrows. The binding site of the DIG-labelled probe is indicated by the black dash. Predicted sizes of the fragments recognized by the probe are indicated. (C) Results of the Southern blot. Chromosomal was digested with ApaLI and separated by electrophoresis. The DNA fragments were subsequently denatured, blotted onto a nylon membrane and hybridized with the DIG-labelled probe. Hybridization was detected using anti-DIG Fab fragments conjugated to alkaline phosphatase. (TIF) [file pgen.1006246.s002.tif]

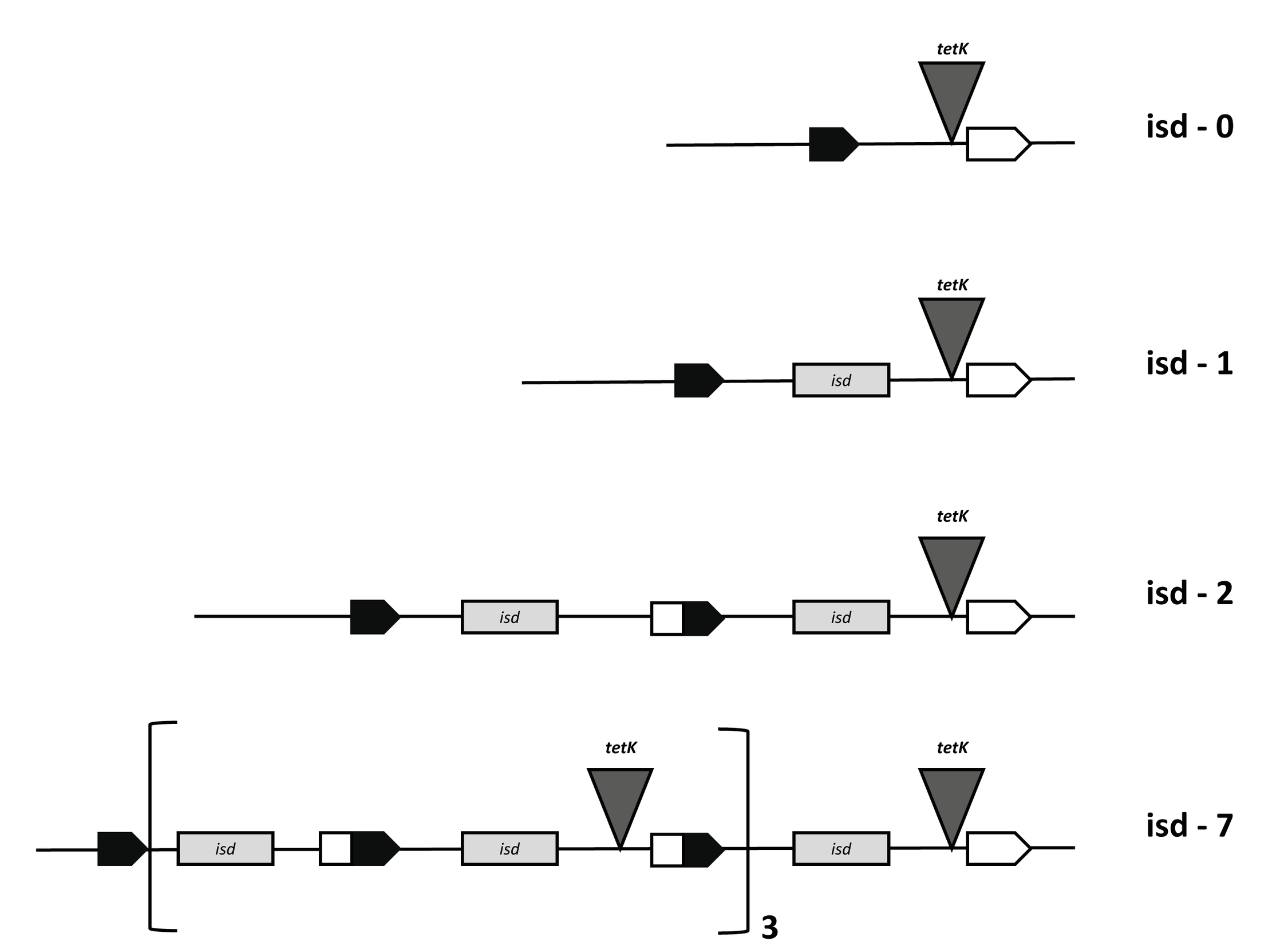

Supplement: S3 Fig — Schematic diagrams of the chromosomal isd-encoding regions in the different strains are shown. Brackets indicate the amplified region in isd—7. (TIF) [file pgen.1006246.s003.tif]

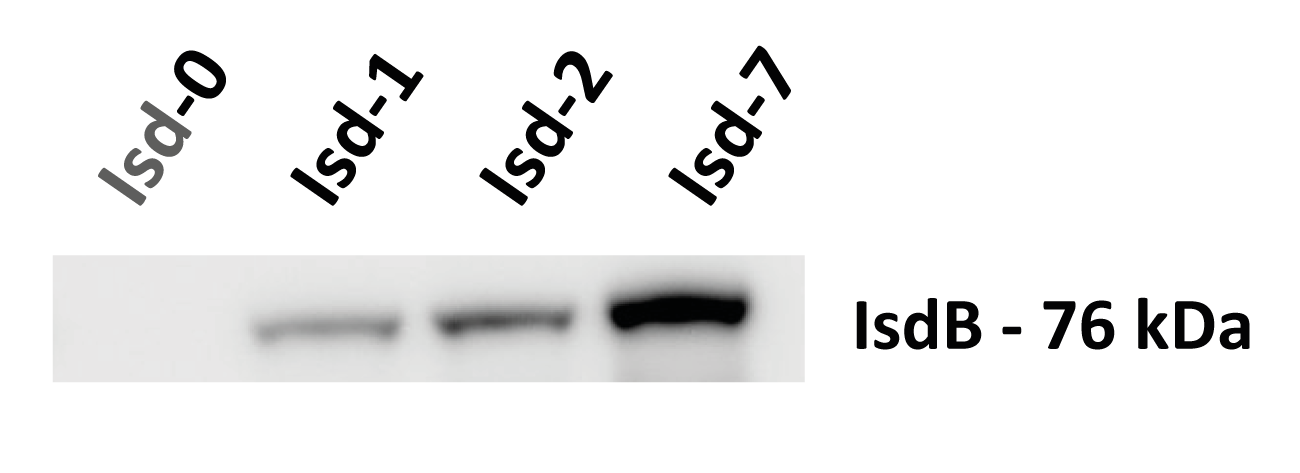

Supplement: S4 Fig — Strains were grown in RPMI, adjusted to OD578 = 5 and the cell wall was digested with lysostaphin and mutanolysin in the presence of 500 mM sucrose to stabilize the protoplasts. Cell wall and membrane fractions were separated by SDS-PAGE and blotted onto a PVDF membrane. Isd proteins were detected using specific rabbit serum followed by goat anti-rabbit IgG conjugated to HRP. IsdB was detected in the cell wall fraction. The experiment was repeated three times. A representative blot is shown. (TIF) [file pgen.1006246.s004.tif]

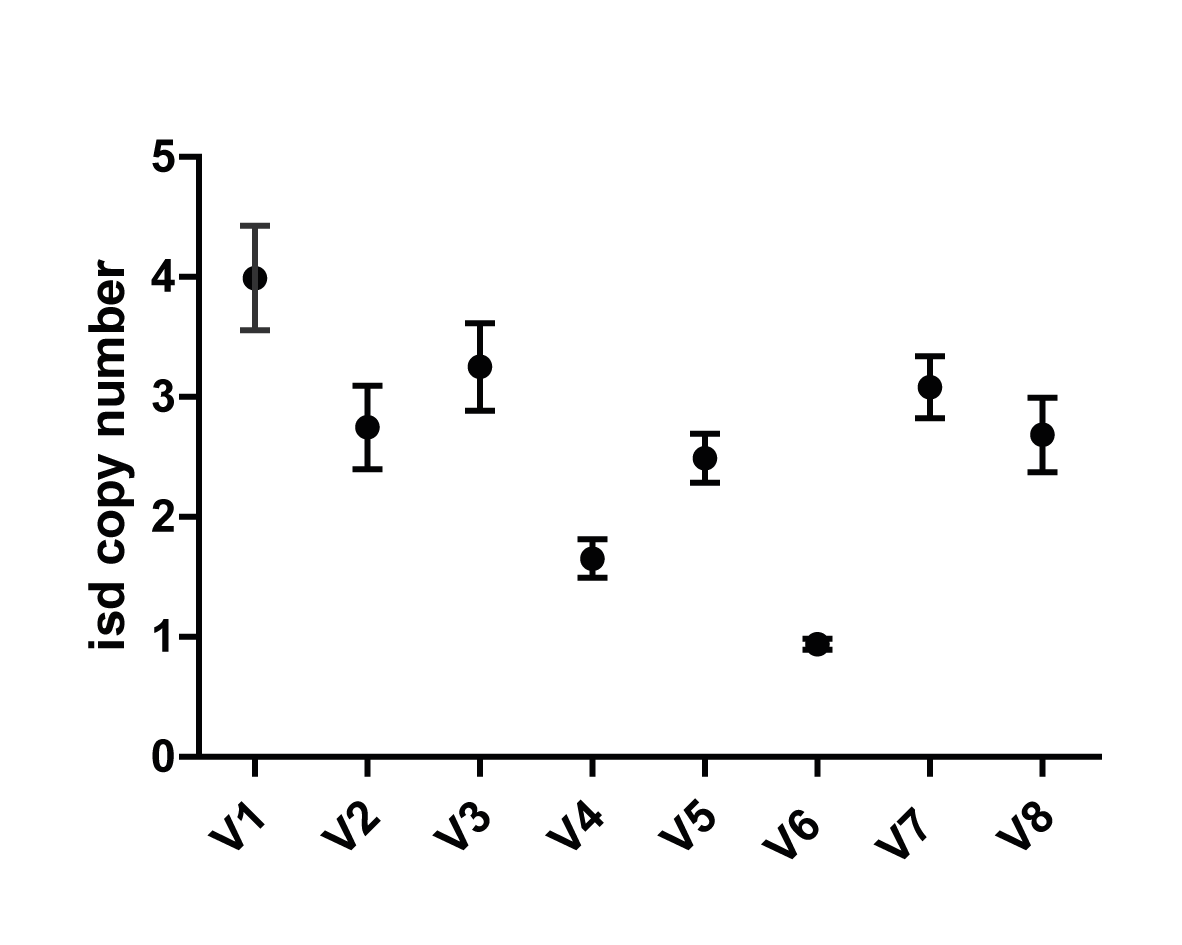

Supplement: S5 Fig — Known concentrations of N920143 DNA (one copy of isd) were used to create the standard curves for isdJ and ori. Relative amounts of template ori and isdJ for each strain were measured. The value for ori was set to 1 and the template amount of isdJ was expressed in relation to this value, thereby giving the copy number of isdJ in the chromosome of each strain. The mean and SD of three experiments is shown. (TIF) [file pgen.1006246.s005.tif]

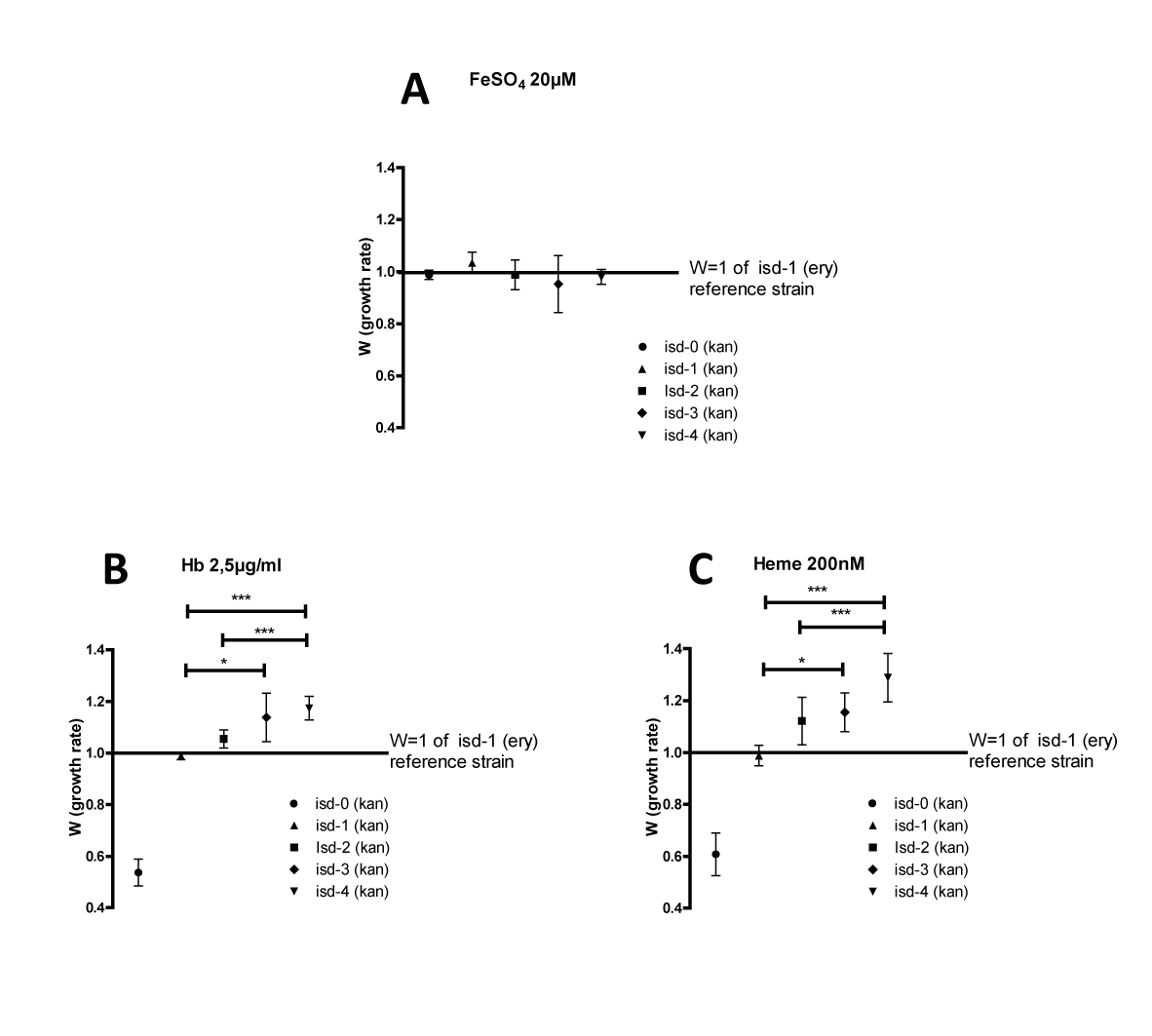

Supplement: S6 Fig — RPMI (with 10μM EDDHA) cultures were supplemented with (A) 20 μM FeSO4, (B) 2,5 μg/ml hemoglobin or (C) 200 nM heme and inoculated with isd-1::pIPI03eryR and one of the isd copy number variants: isd-0::pIPI03KanR, isd-1:: pIPI03KanR, isd-2:: pIPI03KanR, isd-3:: pIPI03KanR, isd-4:: pIPI03KanR. All strains used were RecA positive. The CFU of each strain was enumerated by plating on erythromycin and kanamycin containing agar plates. The change in the ratio of the strains was used to calculate the growth rate of each copy number variant in comparison to the single copy control strain (Wisd-1 was set to 1 for each experiment). The mean and SD of (A) N = 4, (B,C) N = 6 independent experiments is shown. Statistical analysis was performed using One Way Anova followed by Bonferroni’s correction. P-values of <0.05 were regarded as significant and are indicated by*. *** indicate P-values of <0.0001. (TIF) [file pgen.1006246.s006.tif]

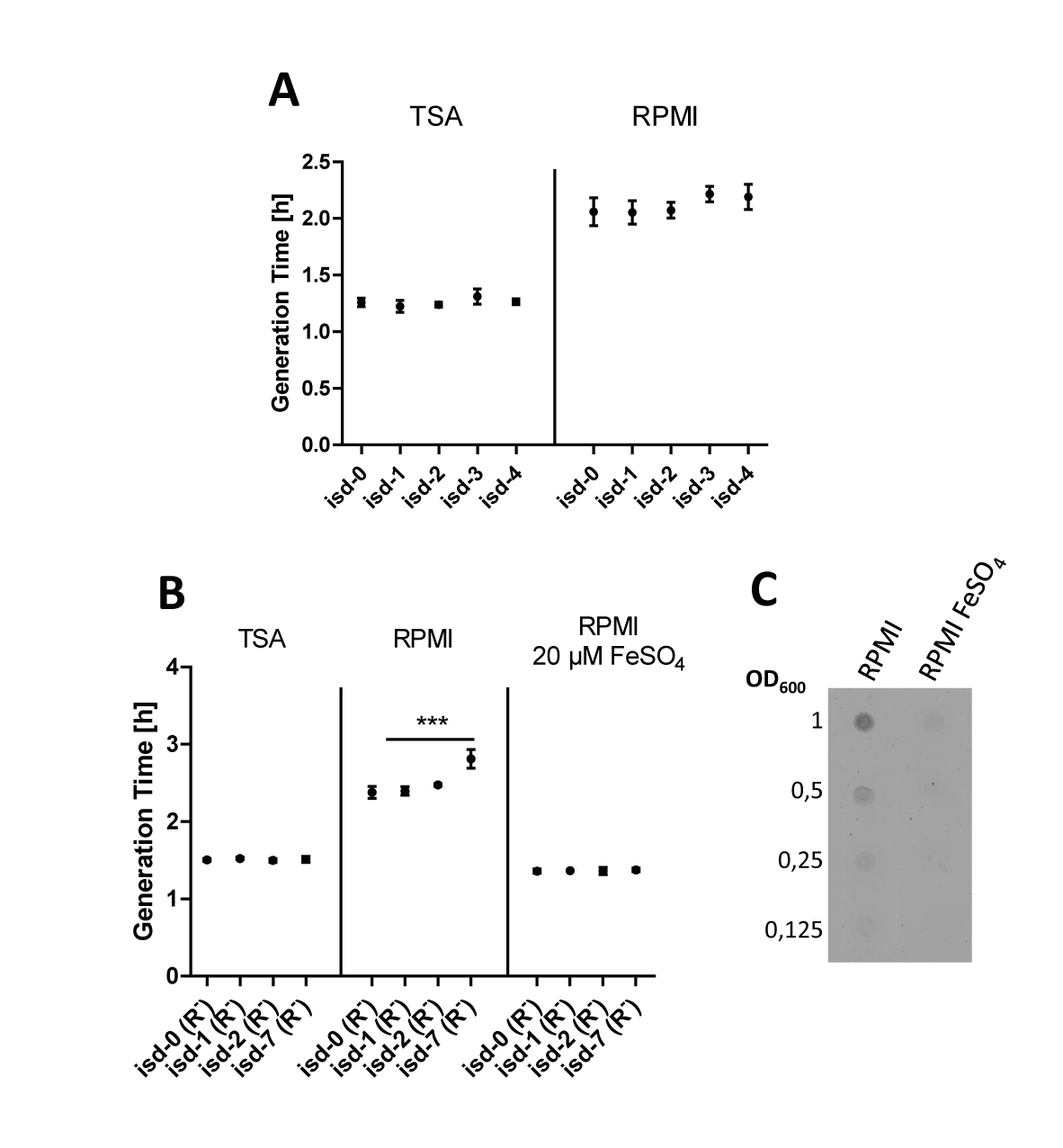

Supplement: S7 Fig — (A) Generation times of RecA positive copy number variants grown in TSB and RPMI. (B) Generation times of RecA negative copy number variant grown in TSB, RPMI and RPMI supplemented with 20 μM FeSO4. Growth assays were performed in 48 well microtiter plates using an Epoch2 reader with 300 rpm shaking at 37°C. OD600nm was determined every 15 minutes. Bacterial generation times were calculated. Shown is the mean and SD of four independent experiments (A) and five independent experiments (B), respectively. Statistical evaluation was performed using One Way Anova followed by Bonferroni’s correction. P-values of <0.05 were regarded as significant and are indicated by*. *** indicate P-values of <0.0001. (C) Whole cell immunoblot: Overnight cultures isd-1 (ΔrecA) were grown overnight in RPMI (with and without 20 μM FeSO4) adjusted to an OD578 = 1 and doubling dilutions were spotted on the membrane. IsdC protein was detected with specific rabbit serum followed by goat anti-rabbit IgG DYLight 800. Fluorescence intensity was measured using Li-Core infrared detection. (TIF) [file pgen.1006246.s007.tif]

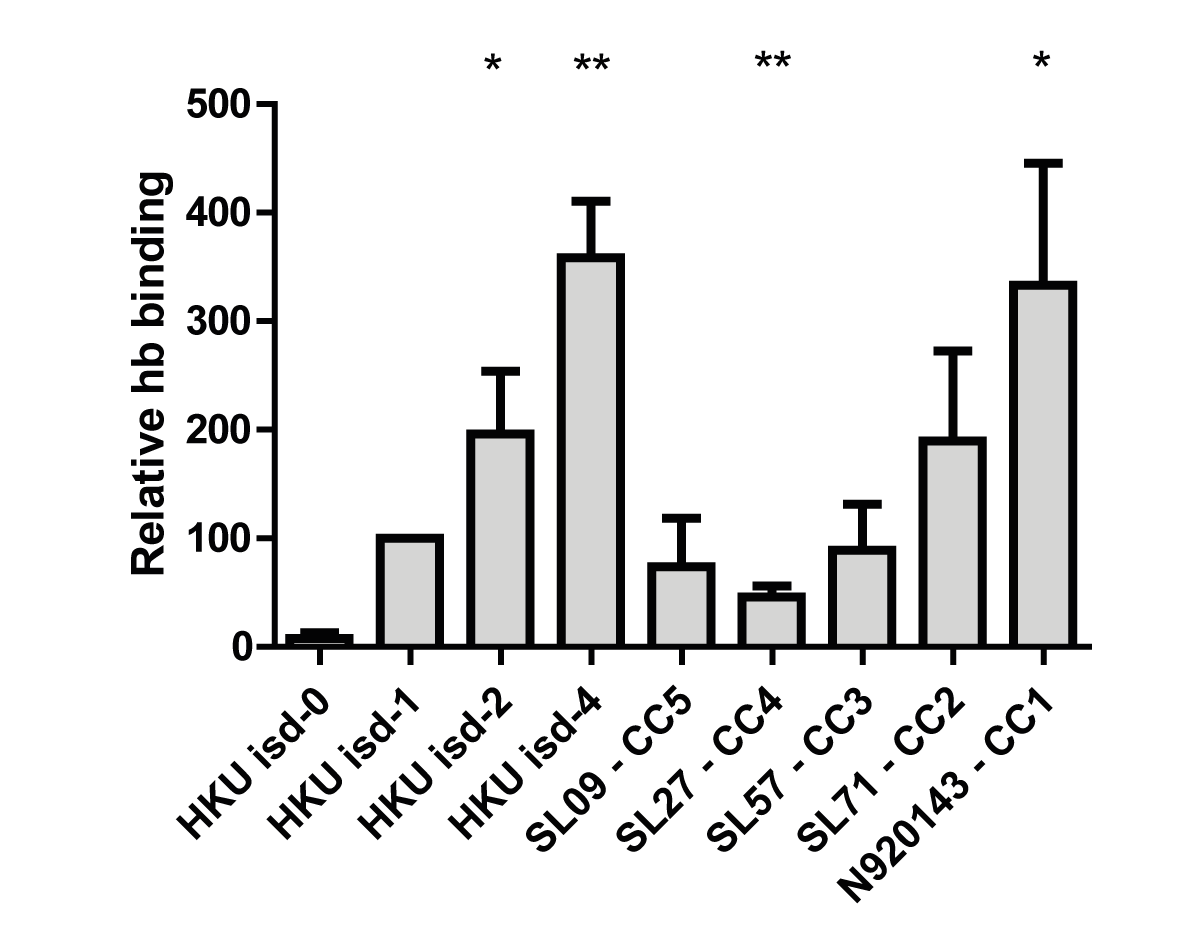

Supplement: S8 Fig — S. lugdunensis strains (all RecA positive) were grown in RPMI with 0,5mM bipiridyl, adjusted to OD578 = 2 and incubated with 10 μg/ml human hemoglobin (hb). After washing cell-surface bound hg was released by boiling and supernatants were separated by SDS-PAGE. Proteins were blotted onto a PVDF membrane. Human hb was detected using specific rabbit serum followed by goat anti-rabbit IgG DYLight 800. Fluorescence intensity was measured quantitatively using Li-Core infrared detection. Absolute values measured for HKU09-01 isd-1 were set to 100% and values obtained for the other strains were expressed in relation to this. The mean and SD of four independent experiments is shown. Strains binding significantly more or significantly less hb than HKU09-01 isd-1 are indicated. Statistical evaluation was performed using a paired two tailed t-test. P-values of <0.05 were regarded as significant and are indicated by*. ** indicate P-values of <0.001. (TIF) [file pgen.1006246.s008.tif]
